# m^6^A modification suppresses ocular melanoma through modulating *HINT2* mRNA translation

**DOI:** 10.1186/s12943-019-1088-x

**Published:** 2019-11-14

**Authors:** Ruobing Jia, Peiwei Chai, Shanzheng Wang, Baofa Sun, Yangfan Xu, Ying Yang, Shengfang Ge, Renbing Jia, Yun-Gui Yang, Xianqun Fan

**Affiliations:** 1 Department of Ophthalmology, Ninth People’s Hospital, Shanghai JiaoTong University School of Medicine, Shanghai, 20025 People’s Republic of China; 2 Key Laboratory of Genomic and Precision Medicine, Collaborative Innovation Center of Genetics and Development, Beijing Institute of Genomics, Chinese Academy of Sciences, Beijing, 100101 China; 3 Shanghai Key Laboratory of Orbital Diseases and Ocular Oncology, Shanghai, 20025 People’s Republic of China; 4 Sino-Danish College, University of Chinese Academy of Sciences, Beijing, 100049 China; 5 Institute for Stem Cell and Regeneration, Chinese Academy of Sciences, Beijing, 100101 China

**Keywords:** m^6^A, Melanoma, *HINT2*, Translation, Tumorigenesis

## Abstract

**Background:**

Dynamic N^6^-methyladenosine (m^6^A) RNA modification generated and erased by N^6^-methyltransferases and demethylases regulates gene expression, alternative splicing and cell fate. Ocular melanoma, comprising uveal melanoma (UM) and conjunctival melanoma (CM), is the most common primary eye tumor in adults and the 2nd most common melanoma. However, the functional role of m^6^A modification in ocular melanoma remains unclear.

**Methods:**

m^6^A assays and survival analysis were used to explore decreased global m^6^A levels, indicating a late stage of ocular melanoma and a poor prognosis. Multiomic analysis of miCLIP-seq, RNA-seq and Label-free MS data revealed that m^6^A RNA modification posttranscriptionally promoted HINT2 expression. RNA immunoprecipitation (RIP)-qPCR and dual luciferase assays revealed that *HINT2* mRNA specifically interacted with YTHDF1. Furthermore, polysome profiling analysis indicated a greater amount of *HINT2* mRNA in the translation pool in ocular melanoma cells with higher m^6^A methylation.

**Results:**

Here, we show that RNA methylation significantly inhibits the progression of UM and CM. Ocular melanoma samples showed decreased m^6^A levels, indicating a poor prognosis. Changes in global m^6^A modification were highly associated with tumor progression in vitro and in vivo. Mechanistically, YTHDF1 promoted the translation of methylated *HINT2* mRNA, a tumor suppressor in ocular melanoma.

**Conclusions:**

Our work uncovers a critical function for m^6^A methylation in ocular melanoma and provides additional insight into the understanding of m^6^A modification.

## Background

N^6^-Methyladenosine (m^6^A), the most prevalent modification in mRNA [1, 2], is a dynamic RNA modification installed by “writer” methyltransferase-like 3 (METTL3), methyltransferase-like 14 (METTL14) and Wilms tumor associated protein (WTAP) [3–5]; erased by “eraser” fat-mass and obesity-associated protein (FTO) and α-ketoglutarate-dependent dioxygenase alkB homolog 5 (ALKBH5) [6, 7]; and recognized by “readers”. Dynamic m^6^A modification affects a variety of cellular processes, such as RNA stability, export, splicing or translation [2]. For instance, m^6^A modification promotes the degradation of *Notch1a* mRNA in the earliest hematopoietic progenitor cells [3] while promoting the translation of immediate-early genes in long-term memory [8]. Therefore, m^6^A RNA modifications have attracted increasing attention in the pathogenesis of human disease.

As m^6^A modifications play a key role in the maintenance of homeostasis, aberrant m^6^A modifications may be an important inducer of tumorigenesis [2]. Disturbance of m^6^A modifications was reported to contribute to the tumorigenesis of glioblastoma, breast cancer and hepatocellular carcinoma [9]. For example, decreased *METTL3* expression or *METTL14* mutation in endometrial cancer reduces the m^6^A modification of AKT pathway-related genes, resulting in the activation of the AKT signaling pathway and contributing to tumorigenesis [10]. In addition, FTO erases m^6^A modification of tumor suppressor genes *MYC/CEBPA*, which contributes to the tumor formation of leukemia. Therapeutically, R-2-hydroxyglutarate inhibits the activity of FTO, thereby suppressing leukemia progression. Thus, exploration of the novel m^6^A RNA methylation drivers of tumorigenesis is potentially interesting.

Ocular melanoma, including uveal melanoma (UM) and conjunctival melanoma (CM), is the most common primary eye tumor in adults and the 2nd most common melanoma, with a high rate of recurrence and poor prognosis [11–13]. The loss of one copy of chromosome 3 has been frequently identified in ocular melanoma. Previous studies have revealed that mutations in G protein subunit alpha Q (GNAQ) or G protein subunit alpha 11 (GNA11) result in the promotion of cell proliferation and sensitize cells to mitogen-activated protein kinase (MAPK) inhibitors. Furthermore, epigenetic drivers, such as DNA methylation, histone modifications, microRNAs and lncRNAs, also participate in tumorigenesis of ocular melanoma [14–16]. For instance, lncRNA *ROR* serves as a decoy oncoRNA that blocks G9a (a key enzyme of histone methylation) binding to the surfaces of target DNA, thereby promoting UM tumorigenesis, while lncRNA CASC15-New-Transcript 1 (*CANT1*) inhibits UM progression by simultaneously activating other lncRNAs, *JPX* and *FTX*. However, the functional role of dynamic tuning of m^6^A in ocular melanoma tumorigenesis remains unclear.

We thus aimed to identify the functional role of m^6^A methylation in malignant ocular melanoma and reveal its potential mechanism in tumorigenesis. We show that m^6^A methylation significantly inhibits the progression of ocular melanoma cells. Mechanistically, m^6^A methylation recognized by YTH N^6^-methyladenosine RNA binding protein 1 (YTHDF1) promotes translation of histidine triad nucleotide-binding protein 2 (*HINT2*), a tumor suppressor in ocular melanoma. Our study reveals the functional importance of RNA m^6^A methylation and thereby presents a novel dynamic mechanism of m^6^A RNA modifications.

## Methods

### Patient samples

A total of 88 human ocular melanoma tissues and 28 human normal melanocyte tissues were collected for immunofluorescence (IF) from patients of Ninth People’s Hospital, Shanghai JiaoTong University School of Medicine from 2007 to 2017. The histological features of all specimens were evaluated by pathologists according to the standard criteria, and the clinic pathological characteristics of ocular melanoma patients are listed in Additional file 2: Table S2 and Additional file 3: Table S3.

### m^6^A RNA methylation assay

Total RNA was extracted from samples using an EZ-press RNA Purification Kit (B0004). The change in m^6^A level relative to total mRNA was measured using the m^6^A RNA Methylation Quantification Kit (Colorimetric) (ab185912) following the manufacturer’s protocol. Each sample was analyzed using 200 ng of RNA isolated from different cells.

### Cell lines

The PIG1 human normal melanocyte cell line was kindly provided by the Department of Ophthalmology, Peking University Third Hospital. The human ocular melanoma cell lines, OCM1, OCM1a and OM431, were kindly supplied by Professor John F. Marshall (Tumor Biology Laboratory, Cancer Research UK Clinical Center, John Vane Science Centre, London, UK). The human conjunctival melanoma cell lines, CRMM1 and CM2005.1, were kindly supplied by Prof. Martine J. Jager (Leiden University Medical Center, Leiden, The Netherlands). The HEK293T human embryonic kidney cell line was purchased from the American Type Culture Collection (Manassas, VA, USA). The cell lines used in this study were authenticated by STR profiling.

### Cell culture

Human OCM1, OCM1a, OM431 and HEK293T cells were cultured in DMEM (GIBCO). PIG1 and CM2005.1 cells were cultured in RPMI 1640 medium (GIBCO). CRMM1 cells were cultured in Ham’s F-12 K (Kaighn’s) Medium (GIBCO). All mediums are supplemented with 10% certified heat-inactivated fetal bovine serum (FBS; GIBCO), penicillin (100 U/mL), and streptomycin (100 mg/mL) and cells are all cultured at 37 °C in a humidified 5% CO2 atmosphere.

### RNA isolation and quantitative real-time PCR

Total RNA was extracted from samples using the EZ-press RNA Purification Kit (B0004), and cDNA was generated using the PrimeScript RT Reagent Kit (Takara). Quantitative real-time PCR using Powerup SYBR Green PCR Master Mix (Life Technologies) was performed using a real-time PCR system (Applied Biosystems).

### Western blot analysis

Cells were harvested at the indicated times and rinsed three times with PBS. Cell extracts were prepared with lysis buffer and centrifuged at 13,000 xg for 30 min at 4 °C. Protein samples were separated by 7.5% (wt/vol) sodium dodecyl sulfate–polyacrylamide gel electrophoresis (SDS-PAGE) and transferred to polyvinylidene fluoride membranes. After blocking with 5% milk for 1 h at room temperature, the membrane was incubated with 2.5 μg/mL of antibody in 5% BSA overnight at 4 °C. The membrane was then incubated with a secondary antibody conjugated to a fluorescent tag (Invitrogen). The band signals were visualized and quantified using the Odyssey Infrared Imaging System (LI-COR, USA).

### Plasmid construction

pLKO.1, pCDH and pCMV were used in our study. ShRNA sequences were generated by PCR and then cloned into the pLKO.1 vector. The *HINT2* overexpression cassette was generated by PCR and cloned into the pCDH vector and verified by DNA sequencing. The *YTHDF1* overexpression cassette was generated by PCR and cloned into the pCMV vector and verified by DNA sequencing.

### Lentivirus packaging and generation of stable cell lines

Lipofectamine 3000 reagent (Invitrogen) was incubated with Opti-MEM I Reduced Serum Medium (GIBCO), and HEK239T cells were transfected with 3 mg of plasmid or 6.0 mg of the PsPax plasmid. Eight hours after transfection, the medium was replaced with 10 mL of fresh medium. The supernatant containing the viruses was collected at 48 and 72 h, filtered through a 0.45-mm cellulose acetate filter and used immediately. Viruses carrying a given plasmid were premixed 1:1, and 50 μL of virus was added to 1 mL of serum. Twenty-four hours prior to transfection, tumor cells were seeded at 2.0 × 10^5^ cells per well in a 6-cm dish, and the medium was replaced with virus-containing supernatant supplemented with 10 ng/mL polybrene (Sigma-Aldrich). After 48 h, the medium was replaced with fresh medium. Cells were selected by incubation with 4 mg/mL puromycin (InvivoGen) for 2 weeks and maintained in 1 mg/mL puromycin (InvivoGen).

### Cell proliferation/growth assays

Cell proliferation/growth was assessed by CCK8 assays (HY-K0301, MCE) following the manufacturer’s instructions. Briefly, cells were seeded in triplicate in 96-well plates at a density of 2000–10,000 cells/100 mL. Dye solution was added at the indicated time points, and the plates were incubated at 37 °C for 3–4 h before the absorbance was detected at 570 nm.

### Apoptosis assays

FITC-Annexin V Apoptosis Detection Kit 1 (BD Biosciences, San Diego, CA) was used following the manufacturer’s instructions. Briefly, cells were washed twice with cold PBS, stained with FITC-Annexin V and PI on ice for 5 min, and subjected to flow cytometric analysis using a BD LSRFortessa analyzer (BD Biosciences).

### Colony formation assay

A volume of 1 mL of complete medium containing 1000 cells was placed in each well of a six-well plate. The plate was stained with 0.25% crystal violet after 1–2 weeks.

### Transwell assay

A 24-well transwell system (Corning) with polycarbonate filters (8-μm pores, Corning) was used. The upper compartment contained 10,000 cells suspended in the appropriate medium with 2% FBS; the lower chamber contained 10% FBS. After 1 day of incubation at 37 °C, the cells in the transwell system were stained with 0.25% crystal violet. The cells remaining in the upper transwell chamber were removed, and those that migrated to the lower chamber were photographed and counted.

### Cell cycle analysis

A sample of approximately 10^6^ cells was centrifuged at 800 xg for 4 min, washed twice with PBS, resuspended in 1 mL of PBS and fixed in 75% ethanol overnight at − 20 °C. The fixed cells were washed three times with 10 mL of ice-cold PBS, resuspended in 200–400 mL of PBS containing 10 mL of RNase (Qiagen) and incubated at 37 °C for 30 min in the dark. The cells were then subjected to FACS at the Flow Cytometry Facility.

### RNA extraction, library construction, Illumina sequencing (RNA-seq) and data analysis

Total RNA was extracted from samples using the EZ-press RNA Purification Kit (B0004). We confirmed the integrity of the RNA using the 2100 Bioanalyzer (Agilent Technologies, USA) and measured the RNA concentration using a Qubit 2.0 fluorometer with a Qubit RNA Assay Kit (Life Technologies, Carlsbad, CA, USA). We then prepared libraries from 100 ng of total RNA using the Illumina TruSeq RNA Sample Prep Kit (San Diego, CA, USA) following the manufacturer’s protocol.

### Methylated RNA immunoprecipitation sequencing (MeRIP-seq)

MeRIP was performed as previously described [17]. Briefly, purified mRNA was randomly fragmented to approximately 100 nucleotides using Ambion RNA fragmentation reagents and then subjected to IP with an anti-m^6^A antibody (202,003, Synaptic Systems) and protein A magnetic beads (88,845, Pierce) in MeRIP buffer (150 mM NaCl, 10 mM Tris-HCl, pH 7.4, 0.1% NP-40) supplemented with RNase inhibitor. m^6^A-containing mRNA fragments were eluted with m^6^A in MeRIP buffer and purified using TRIzol reagent. For MeRIP-seq, two sets of samples were collected for duplicate biological repeats. For MeRIP-qRT-PCR, the same procedures were followed, except that the purified mRNA was fragmented to approximately 200 nucleotides; three biological repeats were conducted. The samples were sequenced with an Illumina HiSeq 4000 platform.

### Cross-linking and methylated RNA immunoprecipitation (miCLIP) -SMARTer-m^6^A-seq

Small-scale, single base-resolution m^6^A methylome detection was carried out following procedures modified from a previous report. Briefly, 100 ng of mRNA was isolated from the tumor (OCM1, OCM1a, OM431, CRMM1 and CM2005.1) and normal (PIG1) cell lines using the Dynabeads mRNA Purification Kit (Life Technologies, 61,006), fragmented to ~ 100 nucleotides using fragmentation reagent (Life Technologies, AM8740), and incubated with 5 μg of an anti-m^6^A antibody (Abcam, ab151230) in 450 μL of IP buffer (50 mM Tris, 100 mM NaCl, 0.05% NP-40, adjusted to pH 7.4) under gentle rotation at 4 °C for 2 h. The mixture was transferred to a clear flat-bottom 96-well plate (Corning) on ice and irradiated three times with 0.15 J/cm^2^ at 254 nm using a CL-1000 Ultraviolet Crosslinker (UVP). After irradiation, the mixture was collected and incubated with 50 μL of prewashed Dynabeads Protein A (Life Technologies, 1001D) at 4 °C for 2 h. After extensive washing twice with high-salt buffer (50 mM Tris, 1 M NaCl, 1 mM EDTA, 1% NP-40, 0.1% SDS, adjusted to pH 7.4) and twice with IP buffer, the samples on the beads were subjected to dephosphorylation with T4 PNK (NEB, M0201 L) at 37 °C for 20 min. The RNA was then eluted from the beads by proteinase K (Sigma, P2308) treatment at 55 °C for 1 h, followed by phenol-chloroform extraction and ethanol precipitation. The purified RNA was subjected to library construction using the SMARTer smRNA-Seq Kit for Illumina (Clontech Laboratories, 635,030) according to the manufacturer’s instructions and sequenced using the Illumina HiSeq X Ten platform.

### RNA-binding protein immunoprecipitation (RIP)-qPCR

To examine m^6^A modification or RNA-binding proteins on individual genes, the Magna RIP™ Quad RNA-Binding Protein Immunoprecipitation Kit (17–704, Millipore, Billerica, MA) was used according to the manufacturer’s instructions. Briefly, 200 mg of total RNA was enriched with antibody- or rabbit IgG-conjugated Protein A/G Magnetic Beads in 500 mL of 1x IP buffer supplemented with RNase inhibitors at 4 °C overnight. RNA of interest was immunoprecipitated with the beads. One-tenth of each fragmented RNA sample was saved as the input control and further analyzed by qPCR.

### Sequencing data analysis

For all untreated tumor and normal cell lines, RNA-seq generated paired-end reads with a length of 151 bp, and MeRIP-seq generated single-end reads with a length of 151 bp. Cutadapt software (version 1.18) [18] was used to trim off the adapter sequences for all raw reads. Reads that contained an ambiguous nucleotide or with lengths less than 18 nt were discarded by Trimmomatic (version 0.36) [19].

The remaining reads were aligned to the human genome (version hg19) using Hisat2 (version 2.1.0) [20]. Only uniquely mapped reads with a mapping quality score ≥ 20 were kept for the subsequent analysis for each sample.

For MeRIP-seq, MACS2 software (version 2.0.10) [21] was used for m^6^A peak calling in each MeRIP sample with the corresponding input sample serving as a control. The default options expected for ‘-nomodel, -keep-dul all’ to turn off fragment size estimation and to keep all uniquely mapped reads in MACS2 were set. Software BEDTools’ intersectBed (version 2.27.1) [22] was used to annotate each peak on the Ensembl (release 72) gene annotation information.

For RNA-seq, the number of reads mapped to each Ensembl (release 72) gene was counted using the software featureCounts (version 1.6.3) [23] from Subread package. FeatureCounts was applied with default options expect for ‘-s 2, -p’ to inform strand-specific library construction and fragment counting for paired-end reads.

### Statistical analysis of differentially expressed genes and genes with differential protein levels

Differentially expressed genes between normal (PIG1) and tumor (OCM1, OCM1a, OM431, CRMM1 and CM2005.1) cell lines were determined using the R-package DEseq2 [24]. Transcripts with a fold change cutoff > 1.5 or < − 1.5 and a *p*-value cutoff < 0.05 were considered significantly differentially expressed genes. Genes with differential protein levels were also determined with the same cutoffs as above.

### Gene ontology analysis

Gene Ontology (GO) analysis of specific genes was performed using DAVID (http://david.abcc. ncifcrf.gov/). GO terms with *P* < 0.05 were statistically significant. Enrichment maps (Fig. 3e, Fig. 4b) were constructed using Cytoscape 3.7.0 installed with the Enrichment Map plugin. Within the enrichment maps, each node represents a GO pathway, and the node size is proportional to the total number of genes in each pathway. The edge thickness represents the number of overlapping genes between nodes. GO pathways of similar functions are sorted into one group, marked with labels and cycles. The number of genes in each cluster is labeled [3].

### Analysis of miCLIP sequencing data

As previously reported [25], raw read preprocessing was performed. Fastx_clipper from fastx_toolkit (http://hannonlab.cshl.edu/fastx_toolkit) was used to trim off the adapter sequence of the raw reads. Fastq_filter.pl, a Perl script from the CLIP Tool Kit (CTK) [26] was used to filter out the low-quality bases, and reads with lengths shorter than 18 nt were discarded. We processed the paired-end data according to the previously reported approach [25] with the same criteria. For individual replicates, we demultiplexed the forward reads based on 5′ barcodes by performing fastq2 collapse to remove PCR-amplified reads, and we reverse complemented the reverse reads and processed them in the same way as the forward counterparts. Finally, stripBarcode.pl was performed to strip the random barcodes of the remaining reads and move to read headers for subsequent analysis processed by the CIMS pipeline.

BWA software (version 0.7.10) [27] was used to map the remaining reads to the human genome (version hg19), and an error rate (substitutions, insertions, or deletions) of ≤0.06 per read was allowed by setting parameter ‘bwa aln –n 0.06 –q20’, following the CTK Online Documentation (https://zhanglab.c2b2.columbia.edu/index.php/CTK_Documentation). The mode of mutation calling was performed as previously reported with minor modifications [28] to identify the m^6^A locus. Program CIMS.pl [29] was used to determine the coverage of the tag number (k) and mutations (m) for each mutation position. The mutation position with m > 1, m/k ≥ 0.01 and m/k ≤ 0.5 were kept, and only mutation positions within the RRACH motif were determined as m^6^A for the subsequent analysis to remove the potential m^6^Am modification [28, 30].

### Motif identification within m^6^A peaks

m^6^A peaks were identified by extending 25 nt both downstream and upstream of the m^6^A sites. The motifs enriched in m^6^A peaks were analyzed by HOMER (version 4.10.3) [31]. Motif length was restricted to 5 nucleotides. The nearby peaks were merged to one peak by performing BEDTools’ merge (version 2.27.1) [22]. These peaks were used as target sequences, and background sequences were constructed by randomly shuffling peaks onto total mRNAs in the genome using BEDTools’ shuffleBed (version 2.27.1) [22].

### Immunofluorescence (IF)

Cells adhering to a glass slide were fixed with 4% formaldehyde (Fisher) for 15 min and then blocked with 5% normal goat serum (Vector) with or without 0.1% Triton X-100 in PBS for 60 min at room temperature. Immunostaining was performed using the appropriate primary and secondary antibodies. Nuclei and the cytoskeleton were counterstained with DAPI and phalloidin, respectively. IF staining was performed with the appropriate Alexa Fluor 488 or Alexa Fluor 546 secondary antibody (Invitrogen, 1:1000 dilution). Images were taken with a ZEISS Axio Scope A1 Upright Microscope.

### RNA pull-down

Biotin-labeled ssRNA probes were synthesized in vitro by Sangon Biotin (Shanghai) Co., Ltd. In vitro RNA-protein pull-down assay were performed using Pierce™ Magnetic RNA-Protein Pull-Down Kit (Thermo Scientific, 20,164) according to the manufacturer’s instructions. The cell lysate was prepared using standard lysis buffers (Thermo Scientific Pierce IP Lysis Buffer) as suggested. 100 pmol of RNA and 50 μL of magnetic beads were used per sample. Input RNA of each sample was mixed with 1 μl 50% glycerol, separated on the 8% native 1x TBE gel, and visualized by phosphorimaging using the Personal Molecular Imager (Bio-Rad) [32–37].

### Luciferase reporter assay

Cells seeded in 6-well plates were transfected with the psiCHECK.2-based luciferase vector fused or not fused to the wild-type or mutated HINT2–3’UTR. Transfection efficiency was quantified by cotransfection with an actin promoter-driven Renilla luciferase reporter. The activities of firefly and Renilla luciferase in each well were calculated using a dual luciferase reporter assay system (Promega). The relative luciferase activity of the HINT2–3’UTR plasmid was further normalized to the signal in cells transfected with the firefly luciferase vector control under the same treatment conditions.

### Polysome profiling

We followed previously reported protocols (https://www.jove.com/pdf/51455/jove-protocol-51455-polysome-fractionation-analysis-mammalian-translatomes-on-genome-wide) with the following modifications. Before collection, 0.1 mg/mL cycloheximide (CHX) was added to the culture medium for 5 min. A sample of 150 million cells from each group was harvested, rinsed in cold PBS with 0.1 mg/mL CHX and quickly frozen in liquid nitrogen before lysis. The lysis buffer was formulated as 10 mM Tris-HCl (pH 7.5), 100 mM NaCl, 30 mM MgCl2, and 0.1 mg/mL CHX with freshly added 1:100 protease inhibitor (Roche) and 40 U/mL SUPERase in RNase Inhibitor (Ambion). The sample was homogenized using a liquid nitrogen grinder. We chose a linear 10 to 40% sucrose gradient according to cell type, and the sample was fractionated into 23 fractions (0.5 mL per fraction) and analyzed using a Gradient Station (BioCamp) equipped with an ECONOUV monitor (BioRad, Hercules, CA) and a Gilson FC203B fraction collector (Mandel Scientific, Guelph, Canada). RNA was purified from fractions 5–22 and subjected to qPCR analysis.

### Mice and animal housing

The animal experiments were approved by the Shanghai Jiao Tong University Animal Care and Use Committee and conducted in accordance with the animal policies of Shanghai JiaoTong University and the guidelines established by the National Health and Family Planning Commission of China. Cells were harvested by trypsinization and washed twice with PBS (GIBCO). BALB/c nude mice (male, 6 weeks old) were used for the study.

### Tumorigenesis assay in vivo

Approximately 1 × 10^6^ melanoma cells from each group were injected subcutaneously into the right side of the abdomen of BALB/c nude mice (male, 6 weeks old). At 30 days after cell injection, the mice were humanely killed, and the tumors were harvested. Each tumor was fixed in 4% formaldehyde, embedded in paraffin, and examined for tumor formation by histologic analysis of hematoxylin and eosin (H&E)-stained sections. Tumor volume was calculated by the formula V = ab^2^/2, where a and b are tumor length and width, respectively.

### Immunohistochemistry (IHC)

Tissue slides were deparaffinized and rehydrated through an alcohol series, followed by antigen retrieval with sodium citrate buffer. Tumor sections were blocked with 5% normal goat serum (Vector) with 0.1% Triton X-100 and 3% H_2_O_2_ in PBS for 60 min at room temperature and then incubated with appropriate primary antibodies at 4 °C overnight. IHC was performed with horseradish peroxidase (HRP) conjugates using DAB detection.

## Results

### Decreased m^6^A in ocular melanoma

**Fig. 1:**
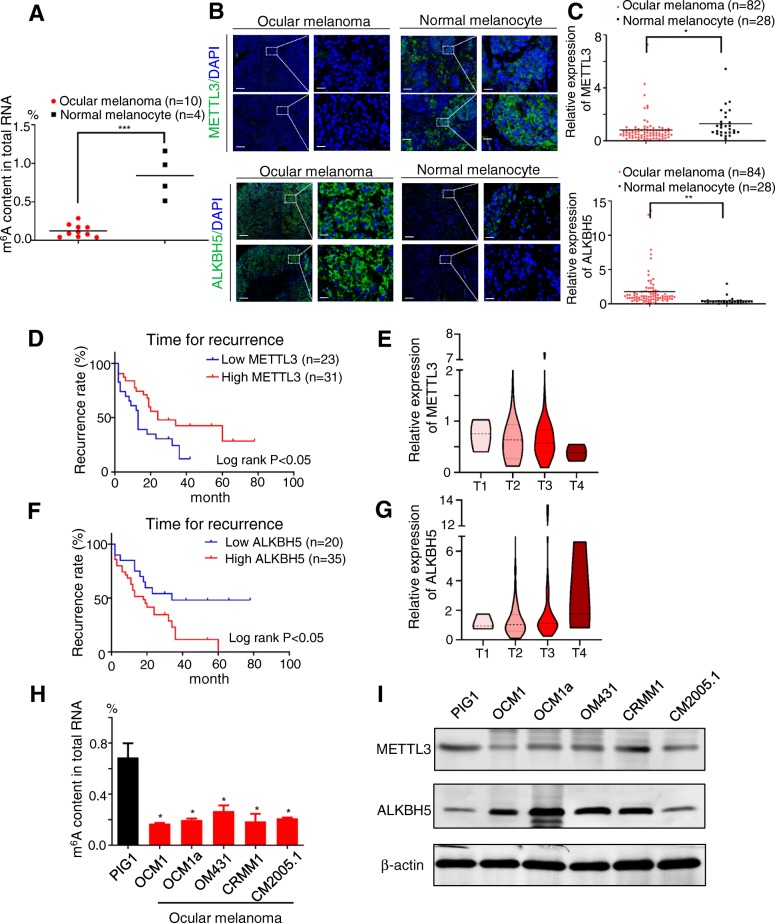
Ocular melanoma exhibited decreased m^6^A levels, which was associated with poor prognosis. **a** An m^6^A RNA methylation assay revealed the m^6^A content in tumor and adjacent normal tissues. For ocular melanoma, *n* = 10, for normal melanocyte, *n* = 4, ***p < 0.001. **b**, **c** METTL3 and ALKBH5 expression in normal and tumor tissues. B: METTL3 and ALKBH5 expression according to IF analysis. Scale bar: left panel, 100 μm; right panel, 20 μm. C: Statistical results of METTL3 and ALKBH5 expression in normal and tumor tissues. *p < 0.05, ***p < 0.001. **d** Kaplan-Meier curves of tumor recurrence showing the difference between ocular melanoma patients with low and high METTL3 levels. *n* = 54, log rank test, *p* < 0.05. **e** The expression of METTL3 in patients at AJCC stages T1 to T4. **f** Kaplan-Meier curves of tumor recurrence showing the difference between ocular melanoma patients with low and high ALKBH5 levels. *n* = 55, log rank test, *p* < 0.05. **g** The expression of ALKBH5 in patients at AJCC stages T1 to T4. **h** The m^6^A RNA methylation assay revealed the m^6^A content in PIG1 cells and ocular melanoma cells. Error bars indicate the mean ± SEM, *n* = 3, *p < 0.05. **i** Western blot showing METTL3 and ALKBH5 expression in normal melanocytes and orbital melanoma cells. The METTL3 signal was quantified and normalized to that of β-actin

To study the functional role of m^6^A methylation in malignant ocular melanoma, we first examined the global m^6^A level in ocular melanoma relative to normal control samples. A significant decrease in m^6^A modification in cellular mRNAs was observed in tumor samples (*p* < 0.001) (Fig. 1a; Additional file 1: Table S1). Notably, the expression of the known m^6^A “writer” METTL3 was also decreased in ocular melanoma tissue compared to normal melanocyte tissue (*p* < 0.05) (Fig. 1b; Additional file 2: Table S2; Additional file 3: Table S3), while the opposite trend was observed for m^6^A “eraser” ALKBH5 (*p* < 0.01) (Fig. 1c; Additional file 2: Table S2; Additional file 3: Table S3). Thus, the decreased m^6^A level in ocular melanoma cells (Fig. 1a) is likely due to the downregulation of METTL3 and upregulation of ALKBH5. Furthermore, lower expression of METTL3 predicted earlier recurrence and enhanced aggressiveness (log rank test, *p* < 0.05) (Fig. 1d, e, Additional file 2: Table S2; Additional file 3: Table S3). Similarly, elevated ALKBH5 expression indicated a poor prognosis (log rank test, *p* < 0.05) (Fig. 1f, g, Additional file 2: Table S2; Additional file 3: Table S3), suggesting that a reduced global m^6^A level predicts higher malignancy. Consistently, we also observed decreased m^6^A modification in ocular melanoma cells (Fig. 1h). Accordingly, decreased METTL3 and upregulated ALKBH5 were observed in ocular melanoma cells (Fig. 1i). These data indicate that a large portion of ocular melanoma is characterized by decreased m^6^A modification, either through decreased METTL3 expression or increased ALKBH5 expression.

### m^6^A methylation inhibits ocular melanoma

**Fig. 2:**
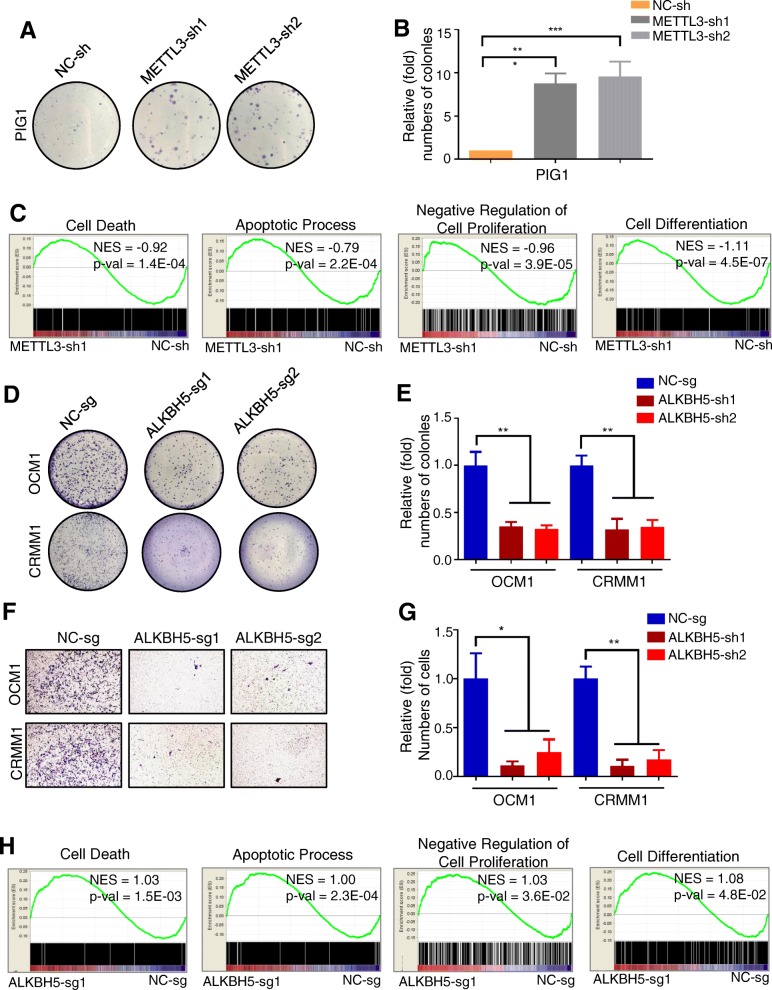
m^6^A methylation inhibited ocular melanoma tumorigenesis. **a** A colony formation assay was performed to assess the tumor growth of PIG1 cells with or without *METTL3* knockdown. **b** Statistics of visible colonies in colony formation assays performed using PIG1 cells with or without *METTL3* knockdown. The colony number of the control group was set to 1. All of the experiments were performed in triplicate, and relative colony formation rates are shown as the mean ± SEM. *n* = 3, ***p < 0.001. **c** GSEA plots evaluating the changes in apoptosis, cell differentiation and cell death in normal melanocyte cells with or without *METTL3* knockdown. NES, normalized enrichment score. **d** A colony formation assay was performed to assess the tumor growth of ocular melanoma cells with or without *ALKBH5* knockdown. **e** Statistics of visible colonies in colony formation assays performed using ocular melanoma cells with or without *ALKBH5* knockdown. The colony number of the control group was set to 1. All of the experiments were performed in triplicate, and relative colony formation rates are shown as the mean ± SEM. *n* = 3*,* **p < 0.01. **f** A transwell assay was performed to evaluate the migratory ability of ocular melanoma cells with or without *ALKBH5* knockdown. **g** Statistics of cells in the transwell assay performed using ocular melanoma cells with or without *ALKBH5* knockdown. The value obtained for the control group was set to 1. All of the experiments were performed in triplicate, and relative metastasis rates are shown as the mean ± SEM. *n* = 3*,* *p < 0.05, **p < 0.01. **h** GSEA plots evaluating the changes in apoptosis, cell differentiation and cell death in ocular melanoma cells with or without *ALKBH5* knockdown. NES, normalized enrichment score

To evaluate whether the global m^6^A level is related to tumorigenesis in ocular melanoma, we first inhibited the expression of methyltransferase *METTL3* in normal pigmented cells and ocular melanoma cells. The *METTL3* expression level decreased to ~ 30% of the original level in normal control cells, PIG1 (Additional file 4: Figure S1A-B), and the global m^6^A level decreased when *METTL3* was knocked down (Additional file 4: Figure S1C), as expected. After silencing *METTL3*, normal melanocyte cells formed an enlarged colony (Fig. 2a, b) with increased growth (Additional file 4: Figure S1D), reduced apoptosis (Additional file 4: Figure S1E), and an increased population in S phase, indicating faster cell division (Additional file 4: Figure S1F). Furthermore, genome-wide gene set enrichment analysis (GSEA) indicated that lower global m^6^A methylation promotes tumorigenesis and identified major regulations of METTL3 (Fig. 2c). Similarly, we silenced the expression of methyltransferase *METTL3* in ocular melanoma. OCM1 and CRMM1 cells were transfected with the same *METTL3* shRNA, and *METTL3* expression levels were successfully knocked down to ~ 20% of the original levels (Additional file 5: Figure S2A-B). Next, we evaluated changes in proliferation and tumorigenesis and found that the colony formation rate increased to almost 200% in melanoma cells with lower m^6^A methylation (Additional file 5: Figure S2C-D). Melanoma cells with *METTL3* knockdown also displayed significantly greater migratory ability (Additional file 5: Figure S2E-F) and slightly accelerated cell growth (Additional file 5: Figure S2G-H).

Because tumor cells present decreased m^6^A modifications, we next inhibited the expression of the demethylase *ALKBH5*. CRISPR/Cas9 is utilized to delete *ALKBH5* in OCM1 and CRMM1 cells (Additional file 6: Figure S3A), and m^6^A methylation increases significantly when *ALKBH5* is knocked down (Additional file 6: Figure S3B). Moreover, the rate of colony formation decreased to 20% in melanoma cells with higher m^6^A methylation (Fig. 2d, e), and these cells showed a significantly weaker migratory ability (Fig. 2f, g). Compared with control cells, ocular melanoma cells with higher m^6^A methylation exhibited significantly decreased tumor growth (Additional file 6: Figure S3C) as more cells underwent apoptosis (Additional file 6: Figure S3D) and fewer cells underwent cell division (Additional file 6: Figure S3E). Genome-wide RNA-seq and GSEA of ocular melanoma cells further demonstrated these antitumor effects of higher m^6^A modification levels and identified major target gene clusters of ALKBH5 (Fig. 2h).

### Transcriptome-wide m^6^A-Seq and RNA-seq assays to identify potential m^6^A-mediated targets

**Fig. 3:**
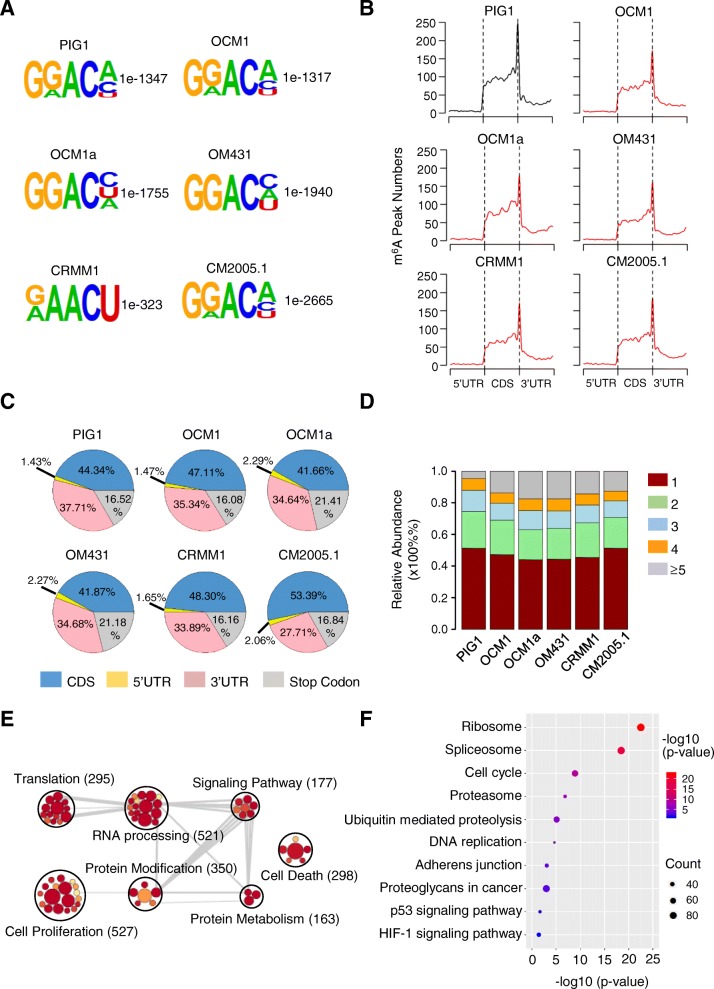
Dynamic m^6^A modifications were highly associated with ocular melanoma tumorigenesis. **a** Top enriched motifs within m^6^A peaks identified in ocular melanoma and normal cells. **b** Distribution of m^6^A sites along the length of mRNA transcripts. **c** Pie charts showing the m^6^A peak distribution in different RNA regions (CDS, 5′ UTR, 3′ UTR and stop codon) in ocular melanoma and normal cells. **d** The percentage of methylated genes with 1, 2, 3, 4 or more than 5 peaks per gene in each cell line. **e** GO enrichment map of m^6^A-containing genes in ocular melanoma and normal cells. **f** KEGG pathway analysis of m^6^A-modified genes in ocular melanoma and normal cells

To understand the regulatory role of m^6^A modification in gene expression, we mapped the m^6^A sites of normal and ocular melanoma cells by MeRIP-seq and m^6^A individual-nucleotide-resolution cross-linking and immunoprecipitation with sequencing (miCLIP-seq), with two independent biological replicates (Additional file 7: Figure S4A). An average of 13,083 and 11,750 m^6^A peaks were identified from miCLIP-seq libraries generated from normal and tumor cells (Additional file 7: Figure S4B). Consistent with previous m^6^A-seq results [3, 4], the m^6^A peaks we identified were characterized by the canonical RRACH motif (Fig. 3a; Additional file 7: Figure S4C) and were enriched in the 3′ UTR, especially near the stop codons (Fig. 3b, c; Additional file 7: Figure S4D-F). Although the motif and the pattern of m^6^A distribution were observed similarly in all cells, we found that the m^6^A modifications were reduced globally in tumor cells compared to normal control cells (Additional file 7: Figure S4G) and that there were more genes with only one m^6^A site in normal cells among the detected m^6^A peaks (Fig. 3d). In total, we identified 5828 genes with significant m^6^A modifications (Additional file 7: Figure S4E). In these genes, terms of cell proliferation and cell death were significantly enriched (Fig. 3e). Moreover, HIF-1 and p53 signaling pathway were significantly enriched to be regulated (Fig. 3f), suggesting a regulation role of m^6^A modification in tumorigenesis.

**Fig. 4:**
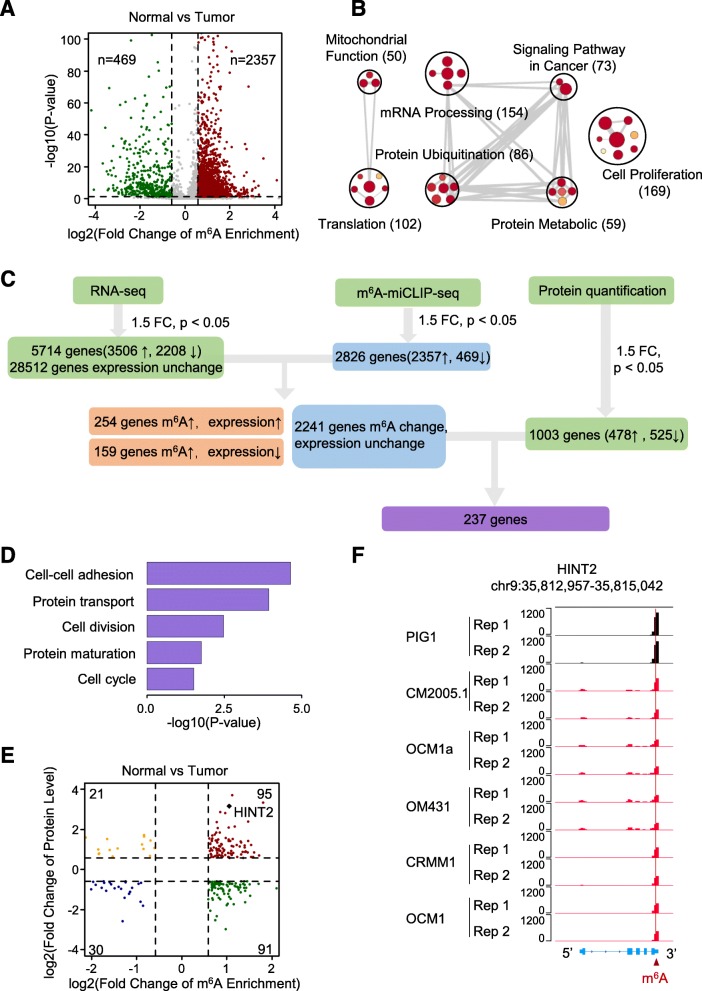
Transcriptome-wide identification of m^6^A targets that regulate tumorigenesis. **a** Volcano plots showing m^6^A enrichment of genes in normal and tumor cells. **b** GO enrichment map of genes with specific enriched m^6^A peaks in control normal cells. **c** Schematic of m^6^A modification downstream analysis. In normal cells, RNA-seq detected 5714 genes with differential expression in which 3506 genes were upregulated and 2208 genes were downregulated. m^6^A-miCLIP-seq showed that 2826 genes were differentially methylated. In normal cells, 2357 genes were up-methylated, and 469 genes were down-methylated. Protein quantification analysis revealed 1003 genes with differential protein levels in normal cells: 478 genes had upregulated protein levels, and 525 genes had downregulated protein levels. In the group of genes with higher m^6^A levels, 254 genes were upregulated, and 159 genes were downregulated. However, 2241 genes with m^6^A methylation levels showed no expression change in normal cells. Finally, we found that 237 genes also had differential m^6^A levels with differential protein levels. The cutoffs used for differentially expressed genes, differential methylation levels and genes with differential protein levels were FC < − 1.5 or FC > 1.5 and *p* < 0.05. FC: fold change, *p*: *p*-value. **d** Representative GO biological process categories enriched in genes with significant differences in m^6^A enrichment and protein content but not gene expression. **e** Volcano plots showing the m^6^A enrichment and protein content of genes in normal cells compared to tumor cells. **f** IGV tracks displaying the miCLIP-seq reads coverage of *HINT2* in normal and tumor cells

In detail, the number of transcripts with greater enriched m^6^A modifications in normal cells was more than 5 times higher than that in tumor cells (Fig. 4a). These transcripts participate in several signaling pathways related to cell proliferation and tumorigenesis (Fig. 4b, Additional file 8: Figure S5A).

To systematically illuminate gene expression changes resulting from the decreased m^6^A modification in ocular melanoma, we used RNA sequencing to compare the transcriptome between normal and tumor cells. Since m^6^A modifications play a key role in the modulation of translation [2], we associatively analyzed the protein expression of genes and, as expected, many genes with m^6^A modifications were inconsistent between mRNA expression and protein expression (Fig. 4c, third panel; Additional file 12: Table S4)**,** suggesting a potential posttranscriptional modulation of translation of m^6^A modifications. Consistently, these genes are involved in the cell cycle, cell adhesion and protein processes (Fig. 4d). *HINT2*, one of the genes with most significant evaluated protein expression and m^6^A enrichment in normal cells, was chosen as a typical example for analyzing the mechanism of m^6^A modifications acting as translation modulators in tumorigenesis (Fig. 4e, Additional file 8: Figure S5C). From our miCLIP-seq data, we identified a statistically significant m^6^A peak in *HINT2* mRNA on the 3’UTR, which was especially enriched in normal cells (Fig. 4f). To confirm our results, we verified decreased m^6^A modifications of *HINT2* mRNA, downregulated expression of HINT2 protein, and unnoticeably altered mRNA levels by RNA-binding protein immunoprecipitation (RIP)-qPCR, Western blotting and qPCR (Additional file 10: Figure S6A-C).

### HINT2 suppresses ocular melanoma in an m^6^A-dependent manner

**Fig. 5:**
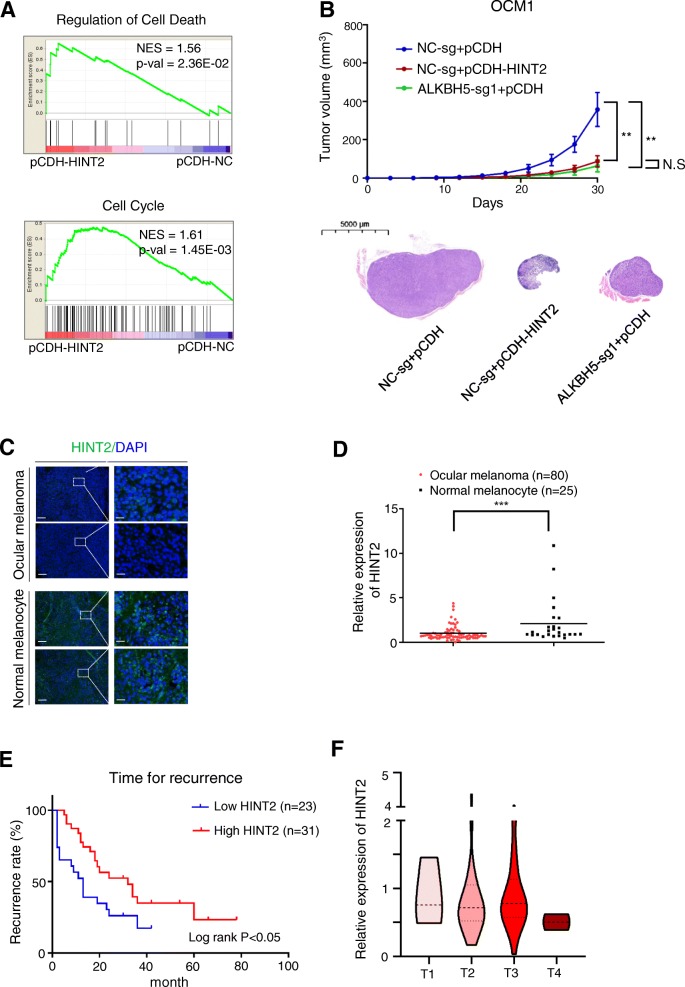
HINT2 acted as a tumor suppressor gene in ocular melanoma cells. **a** GSEA plots evaluating the changes in autophagy, mitochondrial function, apoptosis and cell death in ocular melanoma cells with or without *HINT2* overexpression. NES, normalized enrichment score. **b** The animal experiment shows tumor suppressor roles of *ALKBH5* and *HINT2* in vivo. Top: The line graph shows the volume of subcutaneous tumors formed by the indicated OCM1 cells. Bottom: representative images of H&E staining to evaluate tumor formation. *n =* 5, **p < 0.01; scale bar: 5 mm. **c**, **d** HINT2 expression in normal and tumor tissues. C: HINT2 expression according to IF analysis. Scale bar: top panel, 100 μm; bottom panel, 20 μm. D: Statistical results of HINT2 expression in normal and tumor tissues. *n* = 105, ***p < 0.001. **e** Kaplan-Meier curves of tumor recurrence showing the difference between ocular melanoma patients with low and high HINT2 levels. *n* = 54, log rank test, *p* < 0.05. **f** The expression of HINT2 in patients at AJCC stages T1 to T4

*HINT2* has been reported to act as a tumor suppressor in hepatocellular carcinoma and pancreatic cancer [38, 39], and we confirmed its function in ocular melanoma. We overexpressed *HINT2* in ocular melanoma cells at both the mRNA (Additional file 10: Figure S6D) and protein (Additional file 10: Figure S6E) levels. Notably, cell proliferation (Additional file 10: Figure S6F), cell migration (Additional file 10: Figure S6G-H) and colony formation (Additional file 10: Figure S6I-J) were significantly inhibited and apoptosis was increased (Additional file 10: Figure S6K) in ocular melanoma cells upon *HINT2* overexpression. In addition, GSEA showed that *HINT2* promoted apoptosis and cell death in tumor cells (Fig. 5a). To determine the role of *HINT2* in tumor characteristics in vivo*,* we performed subcutaneous transplantation of ocular melanoma cells with different treatments. We thus tested the tumor suppression role of *HINT2* by overexpressing *HINT2*. In the *HINT2*-overexpressing group (Fig. 5b, Group B) compared with the empty vector group (Fig. 5b, Group A), we found that tumor volume was persistently reduced (*p* < 0.01), meeting the changes created by increased global m^6^A modification (Fig. 5b, Group C). To investigate whether these variations in protein expression occur in samples, we performed IF staining in ocular melanoma and normal tissue and found that HINT2 was indeed downregulated in tumor samples (*p* < 0.001) (Fig. 5c, d). Notably, lower HINT2 expression in ocular melanoma tissue samples was highly correlated with a poor prognosis (log rank test, *p* < 0.05) (Fig. 5e, f; Additional file 2: Table S2; Additional file 3: Table S3).

**Fig. 6:**
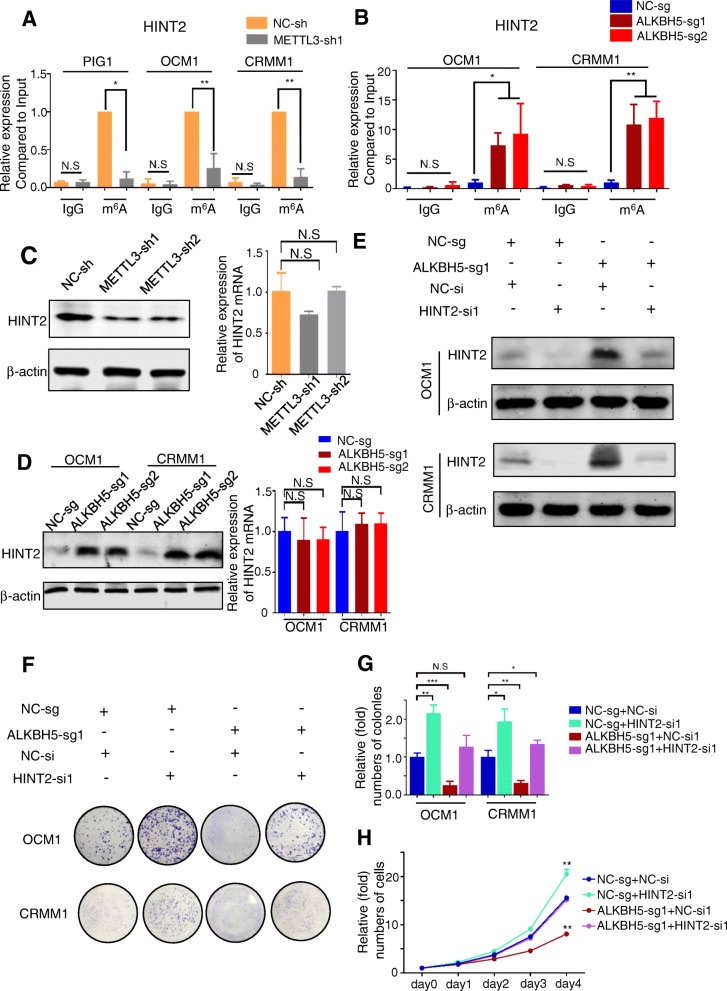
HINT2 protein expression was regulated by m^6^A modification*.* **a** Reduction in m^6^A modification in specific regions of *HINT2* transcripts upon *METTL3* knockdown, as assessed by gene-specific m^6^A-RIP-qPCR assays, in PIG1, OCM1 and CRMM1 cells. The value obtained for the control group was set to 1. Error bars indicate the mean ± SEM, *n* = 3, *p < 0.05, **p < 0.01. **b** Reduction in m^6^A modification in specific regions of *HINT2* transcripts upon *ALKBH5* knockdown, as assessed by gene-specific m^6^A-RIP-qPCR assays, in OCM1 and CRMM1 cells. The value obtained for the control group was set to 1. Error bars indicate the mean ± SEM, *n* = 3, *p < 0.05, **p < 0.01. **c**, **d** Western blot (Left) and real-time PCR (Right) showing *HINT2* expression in PIG1 cells with or without *METTL3* knockdown (**c**) and in OCM1 and CRMM1 cells with or without *ALKBH5* knockdown (**d**). The *HINT2* signal was quantified and normalized to that of *β-actin*. Error bars indicate the mean ± SEM, *n* = 3. **e** HINT2 expression in OCM1 and CRMM1 cells with or without *ALKBH5* knockdown and with or without *HINT2* knockdown. **f** A colony formation assay was performed to assess the tumor growth of OCM1 and CRMM1 cells with or without *ALKBH5* knockdown and with or without *HINT2* knockdown. **g** Statistical analysis of the colony formation assay performed using OCM1 and CRMM1 cells with or without *ALKBH5* knockdown and with or without *HINT2* knockdown. The colony number in the control group was set to 1. All of the experiments were performed in triplicate, and relative colony formation rates are shown as the mean ± SEM. *n* = 3, **p < 0.01, ***p < 0.001. **h** Proliferation of OCM1 and CRMM1 cells with or without *ALKBH5* knockdown and with or without *HINT2* knockdown, as determined by CCK8 assays. *n* = 3, ∗∗*p* < 0.01

Because *HINT2* restrains cell growth and migration in ocular melanoma and shows reduced m^6^A methylation in tumors compared to normal cells, we hypothesized that decreased m^6^A modification might restrain tumor growth partially through upregulating HINT2 expression. The m^6^A status of *HINT2* mRNA was further measured by MeRIP of fragmented RNA, and the m^6^A peak on the 3’UTR was specifically methylated by METTL3 and demethylated by ALKBH5, suggesting functional relevance (Fig. 6a, b). In addition, HINT2 protein expression (Fig. 6c, d, Left), rather than mRNA expression (Fig. 6c, d, Right; Additional file 11 Figure S7A), was decreased when *METTL3* was knocked down, while it was upregulated when *ALKBH5* was knocked down, indicating that *HINT2* translation is positively regulated by its m^6^A modifications. To determine if reduced HINT2 expression underlies the decreased proliferation observed upon enhancing m^6^A modification in ocular melanoma, we attempted to rescue this phenotype by inhibiting *HINT2* expression in upregulated m^6^A cells (Fig. 6e, lanes 3,4). *HINT2* knockdown in *ALKBH5* knockdown cells promoted cell proliferation (Fig. 6f, lanes 3, 4, 6G-H). Importantly, the cell proliferation rates of *ALKBH5* knockdown cells after *HINT2* knockdown were only partially comparable to those of the control cells with normal *ALKBH5* expression (Fig. 6f, lanes 1,4, 6G-H), suggesting that there are more tumor-related genes regulated by their m^6^A modifications. Collectively, m^6^A-guided tumor inhibition partially results from the posttranscriptional regulation of *HINT2*.

### m^6^A modification promotes HINT2 translation

**Fig. 7:**
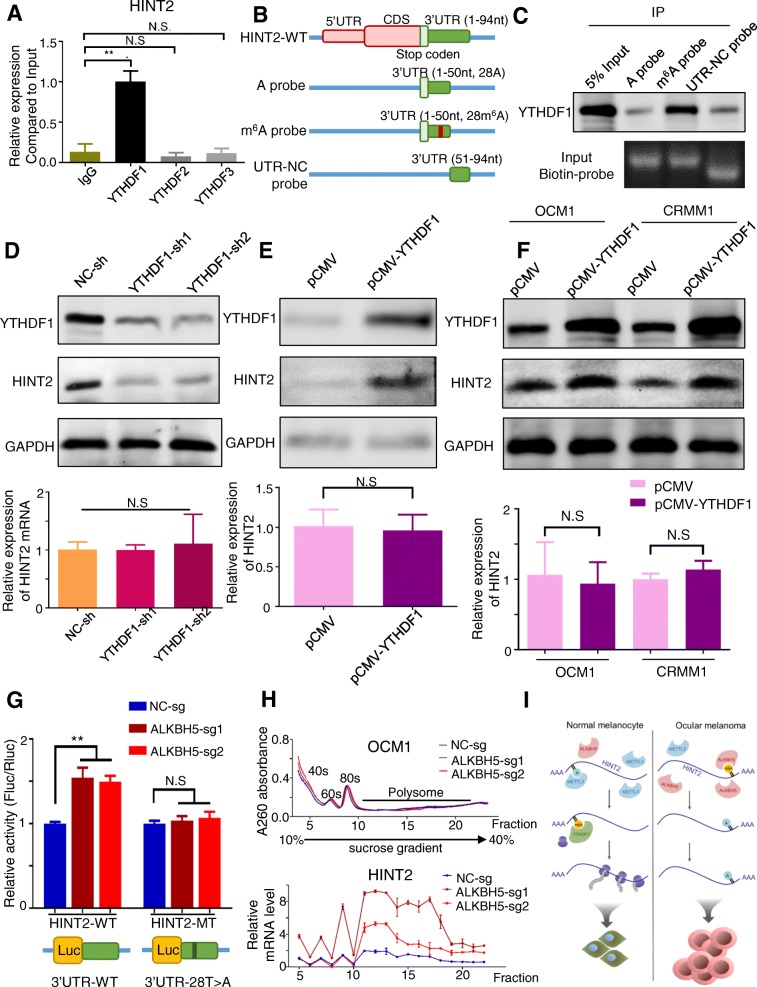
YTHDF1 promoted HINT2 translation*.* **a** Reduction in m^6^A modification at specific regions of *HINT2* transcripts, as assessed by gene-specific YTHDF1, YTHDF2 and YTHDF3 RIP-qPCR assays, in PIG1 cells. The value obtained for the IgG was set to 1. Error bars indicate the mean ± SEM, *n* = 3, **p < 0.01. **b** Model showing RNA probes used in RNA pull-down assays. **c** RNA pulldown of endogenous YTHDF1 proteins from HEK293T nuclear extract using synthetic *HINT2* RNA fragments with or without m^6^A modifications. Images are representative of 3 independent experiments. **d** Western blotting (Top) and real-time PCR (Bottom) were performed to elucidate *HINT2* expression in PIG1 cells with or without *YTHDF1* knockdown. The *HINT2* and *YTHDF1* signals were quantified and normalized to that of *GAPDH*. Error bars indicate the mean ± SEM, *n* = 3. **e**, **f** Western blotting (Top) and real-time PCR (Bottom) were performed to elucidate *HINT2* expression in PIG1 cells with or without *YTHDF1* overexpression. The *HINT2* and *YTHDF1* signals were quantified and normalized to that of *GAPDH*. Error bars indicate the mean ± SEM, *n* = 3. **g** Dual luciferase reporter assays showing the effect of ALKBH5 on *HINT2* mRNA reporters with either wild-type or mutated m^6^A sites. Error bars indicate the mean ± SEM, *n* = 3, ***p < 0.001. **h** Polysome profiling assays. The fractionation of lysates from PIG1 cells with or without *METTL3* knockdown is shown on the top. RNAs in different ribosome fractions were extracted and subjected to qPCR analysis; data are shown on the bottom as the mean ± SD. *n* = 3, *p < 0.05; **p < 0.01; ***p < 0.001. **i** Model showing how reduced m^6^A methylation alters *HINT2* translation to contribute to tumor progression

Because m^6^A methylation appears to promote the translation of *HINT2*, we hypothesized that *HINT2* transcripts are targets of the YTH family, the m^6^A reader that regulates translation of m^6^A modified transcripts [40]. RIP-qPCR analysis revealed that *HINT2* mRNAs interacted with YTHDF1 more strongly than the others (Fig. 7a). In addition, RNA pull-down assays further confirmed that YTHDF1 interacted with *HINT2* mRNA and that the m^6^A modification of *HINT2* greatly facilitated its binding to YTHDF1 (Fig. 7b, c, Additional file 15: Table S5). Consistently, *YTHDF1* knockdown (Fig. 7d line 1, Additional file 11: Figure S7B) decreased HINT2 protein expression (Fig. 7d line 2), while *YTHDF1* overexpression (Fig. 7e, f line 1, Additional file 11: Figure S7C-D) increased HINT2 protein expression (Fig. 7e-F line2). These changes in HINT2 expression are not due to changes in the abundance of the *HINT2* transcript (Fig. 7d-f, Bottom); rather, the regulation of mRNA translation relies on the methylation of transcripts. We created reporter constructs with *HINT2* 3′ UTR (WT) or m^6^A locus mutated (MT) sequences (Additional file 15: Table S5), and dual luciferase assays showed that ALKBH5 significantly decreased the luciferase activity of reporters carrying the wild-type fragment instead of the corresponding mutant fragment (Fig. 7g). In detail, the association of the *HINT2* transcript with actively transcribing ribosomes was improved by its m^6^A modifications (Fig. 7h, Additional file 11: Figure S7E). Altogether, these experiments reveal that m^6^A modification has an impact on the translation of *HINT2* when recognized by YTHDF1.

## Discussion

Here, we discovered that m^6^A mRNA methylation regulates the translation of the tumor suppressor gene *HINT2* and thereby regulates ocular melanoma tumorigenesis (Fig. 7i). One-third of mRNAs are m^6^A modified, which may regulate mRNA stability and translation [2]. A previous study discovered that the “writers” of m^6^A methylation, METTL3 and METTL14, could play both oncogenic and tumor suppressor roles in numerous cancers [40, 41], while oncogenic roles have been reported for “erasers” such as FTO and ALKBH5 [7, 42, 43]. Here, we found that the decreased global m^6^A modification in ocular melanoma promoted tumorigenesis, which resulted from decreased “writers” and upregulated “erasers”. Our study indicates that the disturbance of global m^6^A homeostasis is a key driver in the regulation of tumor formation, which depends on the balance of “writers” and “erasers” of m^6^A modification.

Notably, the balance of “writers” and “erasers” regulates clusters of gene function, and the AKT pathway is one of the most important targets regulated by m^6^A modifications. The expression of some key genes of the AKT pathway, such as *PHLPP2*, *PTEN*, *Bcl2* and *c-Myc*, has been reported to be regulated by their m^6^A modifications. These modifications can be written by METTL3 or METTL14 and can be erased by ALKBH5 and regulate the tumorigenesis of leukemia, pancreatic cancer, endometrial cancer and gastric cancer [10, 30, 44–46]. Although we did not find a significant regulation of the AKT pathway by m^6^A modifications in ocular melanoma, genes in other biological processes, such as mRNA processing, translation, Hippo-YAP signaling and MAPK signaling, were differentially expressed (Fig. 4b, Additional file 8: Figure S5A-C). These signaling pathways have been reported to be involved in m^6^A targets in colorectal cancer and lung cancer and in the inflammatory response [47–49]. Other tumor-related genes, such as *ACAT2*, *PIR* and *CTBP1*, were potentially regulated by m^6^A modifications in ocular melanoma. Further studies should be performed to understand m^6^A modification systematically.

Notably, the regulation mechanisms of m^6^A modifications are complicated, reflected not only in multitudinous targets but also in different regulations in various cells. It should be noted that the key enzyme of m^6^A modification may have opposite effects on tumorigenesis in different systems, even within one specific type of tumor. For example, Somasundaram et al. found that *METTL3* acts as an oncogene that promotes tumorigenesis, glioblastoma stem cell maintenance, and radio resistance by regulating SRY-Box 2 (*SOX2*) [50], whereas Shi et al. reported opposite effects, including inhibition of tumorigenesis and glioblastoma stem cell self-renewal/ proliferation via regulation of ADAM metallopeptidase domain 19 (*ADAM19*) [43]. METTL14 inhibits tumor invasion and metastasis by regulating *miR-126* [51] while promoting tumor cell proliferation and migration by regulating suppressor of cytokine signaling 2 (*SOCS2*) [41]. In addition, the key enzyme of m^6^A modification could also be involved in other non-m^6^A modification activities. For example, METTL3 and YTH N^6^-methyladenosine RNA binding protein 3 (YTHDF3) directly regulate translation by cooperating with eukaryotic translation initiation factor 3 (eIF3), independent of m^6^A [52, 53]. Future systematic studies could focus on the detailed mechanism of m^6^A-related proteins regulating tumor fate.

m^6^A-modified RNA recognized by different readers could present with different functions. For instance, YTHDF1 mediates nuclear export and translation [10, 54], YTH N^6^-methyladenosine RNA binding protein 2 (YTHDF2) regulates mRNA stability with m^6^A [10, 55] and RNA structural remodeling [33], and YT521-B regulates sex-specific alternative splicing and subcellular localization of mRNAs with m^6^A [56, 57]. Here, we revealed for the first time that the m^6^A modification of *HINT2* is recognized by YTHDF1. Further study could concentrate on the oncogenic role of other readers, such as YTHDF2/3, in regulating tumor formation of ocular melanoma.

HINT2 is a member of the superfamily of histidine triad AMP-lysine hydrolase proteins, which are closely associated with mitochondrial metabolism and tumor suppression [58]. HINT2 has been reported to expedite Ca^2+^ influx into mitochondria from the cytoplasm, which is a hallmark of the mitochondrial apoptosis pathway and an essential condition for early apoptosis [58]. Loss of HINT2 disturbs mitochondrial lipid metabolism, glucose homeostasis and mitochondrial deformity [59]. As a tumor suppressor gene, *HINT2* is downregulated in pancreatic cancer and hepatocellular cancer, endometrial cancer and colorectal cancer, preventing the mitochondrial apoptosis pathway and leading to poor survival [38, 39, 60, 61]. Here, we revealed for the first time that *HINT2* is regulated by m^6^A modification, indicating that disturbances in RNA methylation homeostasis result in dysregulation of proliferation and mitochondrial apoptosis, which contributes to the progression of ocular melanoma. Notably, we found that knockdown of *HINT2* only partially rescued the effect of knockdown of *ALKBH5* on ocular melanoma cells, which indicates that there are other cofactors involved in tumorigenesis that are regulated by m^6^A modifications.

Uveal melanoma is the most common adult intraocular tumor, with a 5-year survival rate ranging from 71 to 76% [11]. Additionally, more than half of patients develop metastasis after 5 years [11], and the median survival time for metastatic UM is only 12 months [62]. Conjunctival melanoma is also a rare but lethal cancer; nearly 30% of patients die within 10 years [63]. Although B-raf mutations are rare in UM, activation changes in other MAPK pathway components, such as *GNAQ* or *GNA11*, are found in 85–95% of patients and are partly responsible for tumorigenesis [64, 65]. In CM, the *B-raf* mutation is present in up to 50% of patients [66]. Ocular melanoma cells with the activated MAPK pathway have been reported to be modestly sensitive to MAPK/ERK kinase (MEK) inhibitors with or without combination treatment with the protein kinase C (PKC) inhibitor [67]. Unfortunately, PKC targeting is limited by toxicity, and a completed phase 3 trial showed no clinical benefit [68]. To date, structure-based selective inhibitors of the m^6^A key enzyme have been discovered [69, 70]. These drugs provide a novel pathway for targeted therapy of ocular melanoma.

## Conclusion

In general, we revealed the critical role of m^6^A modification in ocular melanoma tumorigenesis. Decreased m^6^A levels are identified in ocular melanoma samples, indicating poor prognosis, and changes in global m^6^A modification are highly associated with tumor progression. Mechanistically, YTHDF1 promotes the translation of methylated mRNA of *HINT2*, a tumor suppressor in ocular melanoma. Our work uncovers a critical function of m^6^A methylation in ocular melanoma and provides insight into the understanding of m^6^A modification.

## Supplementary information

**Additional file 1: Table S1.** The clinical characteristics of ocular melanoma patient cohorts in the m^6^A assay.

**Additional file 2: Table S2.** The clinical characteristics of ocular melanoma patient cohorts in tissue chip.

**Additional file 3: Table S3.** The detail information of clinical characteristics and gene expression of ocular melanoma patient cohorts in tissue chip.

**Additional file 4: Figure S1.** m^6^A methylation promoted PIG1 cell proliferation.

**Additional file 5: Figure S2.** Lower m^6^A methylation promoted ocular melanoma tumorigenesis.

**Additional file 6: Figure S3.** Higher m^6^A methylation inhibited ocular melanoma tumorigenesis.

**Additional file 7: Figure S4.** m^6^A-seq of ocular melanoma and normal control cells.

**Additional file 8: Figure S5.** Characterization and modulation of m^6^A in ocular melanoma cells*.*

**Additional file 9: Table S4.** List of protein expression of genes in ocular melanoma cells and normal cells according to the Label-free MS.

**Additional file 10: Figure S6.** Overexpression of *HINT2* inhibited ocular melanoma tumorigenesis.

**Additional file 11: Figure S7**. *HINT2* translation was promoted by m^6^A modification.

**Additional file 12: Table S5.** Oligonucleotides used in this study.

**Additional file 13: Table S6.** Antibodies used in this study.

**Additional file 14: Table S7.** Primers used in this study.

**Additional file 15.** Unprocessed original scans of blots.

## Data Availability

The raw sequence data reported in this paper, including RNA-seq, miCLIP-seq and MeRIP-seq data, have been deposited in the Gene Expression Omnibus database under accession number GSE137675, and also the Genome Sequence Archive [71] in the BIG Data Center [72], Beijing Institute of Genomics (BIG), Chinese Academy of Sciences, under accession number CRA001675 (http://bigd.big.ac.cn/gsa/s/n110138p) and are publicly accessible at http://bigd.big.ac.cn/gsa.

## Abbreviations

ADAM19: ADAM metallopeptidase domain 19
ALKBH5: α-ketoglutarate-dependent dioxygenase alkB homolog 5
CANT1: LncRNA CASC15-New-Transcript 1
CM: Conjunctival melanoma
eIF3: Eukaryotic translation initiation factor 3
FTO: Fat-mass and obesity-associated protein
GNA11: G protein subunit alpha 11
GNAQ: G protein subunit alpha Q
GSEA: Genome-wide gene set enrichment analysis
HINT2: Histidine triad nucleotide-binding protein 2
m^1^A: N^1^-methyladenosine
m^5^C: N^5^-methylcytosine
m^6^A: N^6^-methyladenosine
m^6^Am: N^6^,2′-O-dimethyladenosine
MAPK: Mitogen-activated protein kinase
MEK: MAPK/ERK kinase
MeRIP-seq: methylated RNA immunoprecipitation sequencing
METTL14: Methyltransferase-like 14
METTL3: Methyltransferase-like 3
miCLIP-seq: m^6^A individual-nucleotide-resolution cross-linking and immunoprecipitation with sequencing
PKC: Protein kinase C
RIP: RNA-binding protein immunoprecipitation
SOCS2: Suppressor of cytokine signaling 2
SOX2: SRY-box 2
UM: Uveal melanoma
UTR: Untranslated region
WTAP: Wilms tumor associated protein
YTHDF1: YTH N^6^-methyladenosine RNA binding protein 1
YTHDF2: YTH N^6^-methyladenosine RNA binding protein 2
YTHDF3: YTH N^6^-methyladenosine RNA binding protein 3
