# Supplementary material for: m6A modification suppresses ocular melanoma through modulating HINT2 mRNA translation
Source: Mol Cancer. 2019 Nov 14;18:161. doi: 10.1186/s12943-019-1088-x (PMC6854757; doi:10.1186/s12943-019-1088-x)
Supplement: Supplementary file 1 — Additional file 1: Table S1. The clinical characteristics of ocular melanoma patient cohorts in the m6A assay. [file 12943_2019_1088_MOESM1_ESM.pdf]

Additional file 1: **Table S1.** The clinical characteristics of ocular melanoma patient cohorts in the $m^6A$ assay.

| Features | Ocular melanoma | Normal uveal |
|---|---|---|
| Numbers | 10 | 4 |
| Sex, F/M | 6/4 | 1/3 |
| Age | 47.2±19.52 | 17.1±7.66 |
| Stage(AJCC 7$^{th}$) | | |
| T1 | 0 | / |
| T2 | 0 | / |
| T3 | 5 | / |
| T4 | 5 | / |
