# Supplementary material for: m6A modification suppresses ocular melanoma through modulating HINT2 mRNA translation
Source: Mol Cancer. 2019 Nov 14;18:161. doi: 10.1186/s12943-019-1088-x (PMC6854757; doi:10.1186/s12943-019-1088-x)
Supplement: Supplementary file 2 — Additional file 2: Table S2. The clinical characteristics of ocular melanoma patient cohorts in tissue chip. [file 12943_2019_1088_MOESM2_ESM.pdf]

Additional file 2: **Table S2.** The clinical characteristics of ocular melanoma patient cohorts in tissue chip assay.

Features	Ocular melanoma	Normal mylanocyte
Numbers	88	28
Sex, F/M	23/65	18/10
Age	54.91±18.16	39.75±19.91
Stage(AJCC 7th)		
T1	4	/
T2	38	/
T3	43	/
T4	3	/
