# Supplementary figures and images for: m6A modification suppresses ocular melanoma through modulating HINT2 mRNA translation
Source: Mol Cancer. 2019 Nov 14;18:161. doi: 10.1186/s12943-019-1088-x (PMC6854757; doi:10.1186/s12943-019-1088-x)

Additional file 4: **Figure S1.**

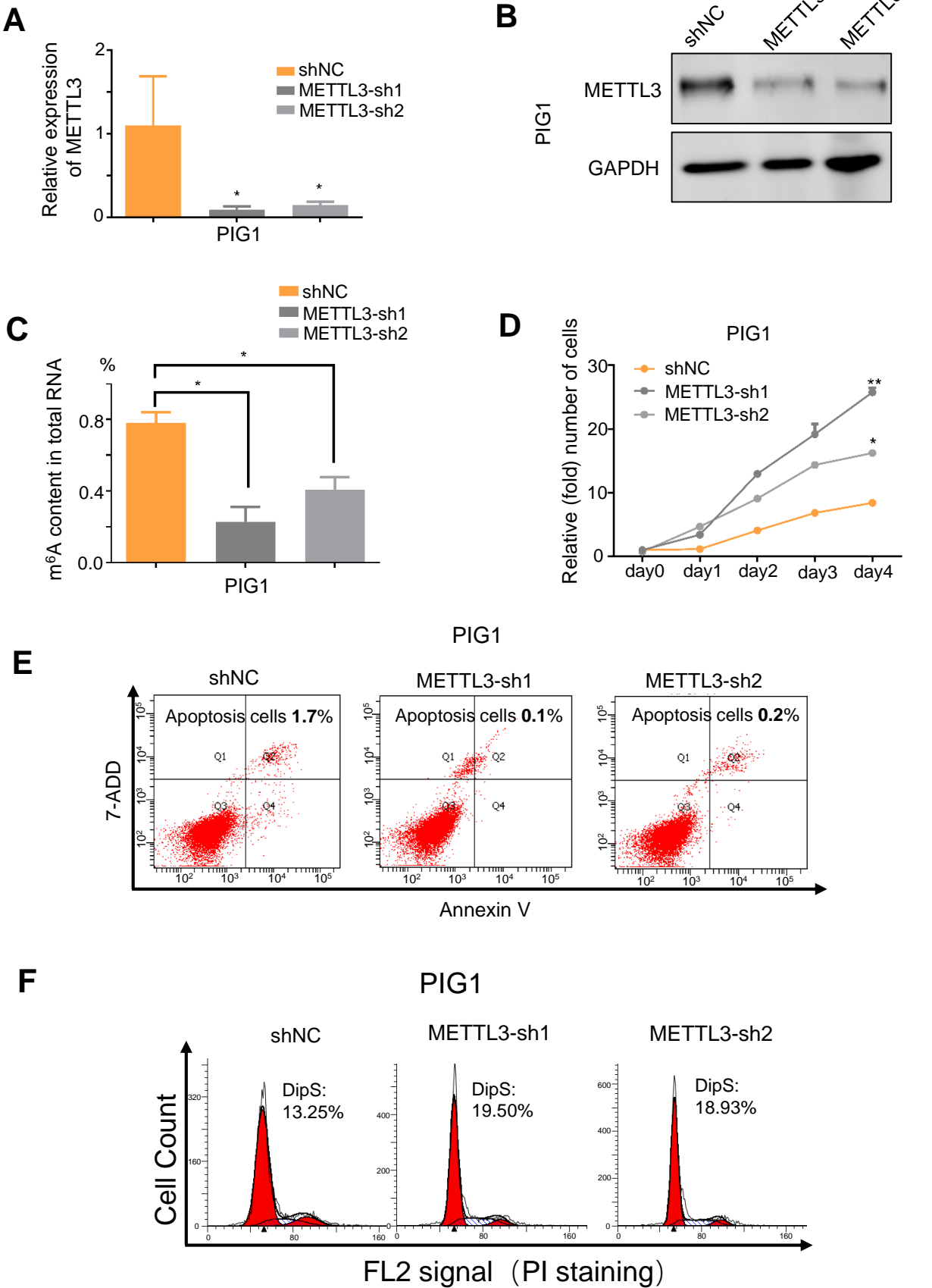

Supplement: Supplementary file 4 — Additional file 4: Figure S1. m6A methylation promoted PIG1 cell proliferation. [file 12943_2019_1088_MOESM4_ESM.pdf]

# Additional file 5: Figure S2

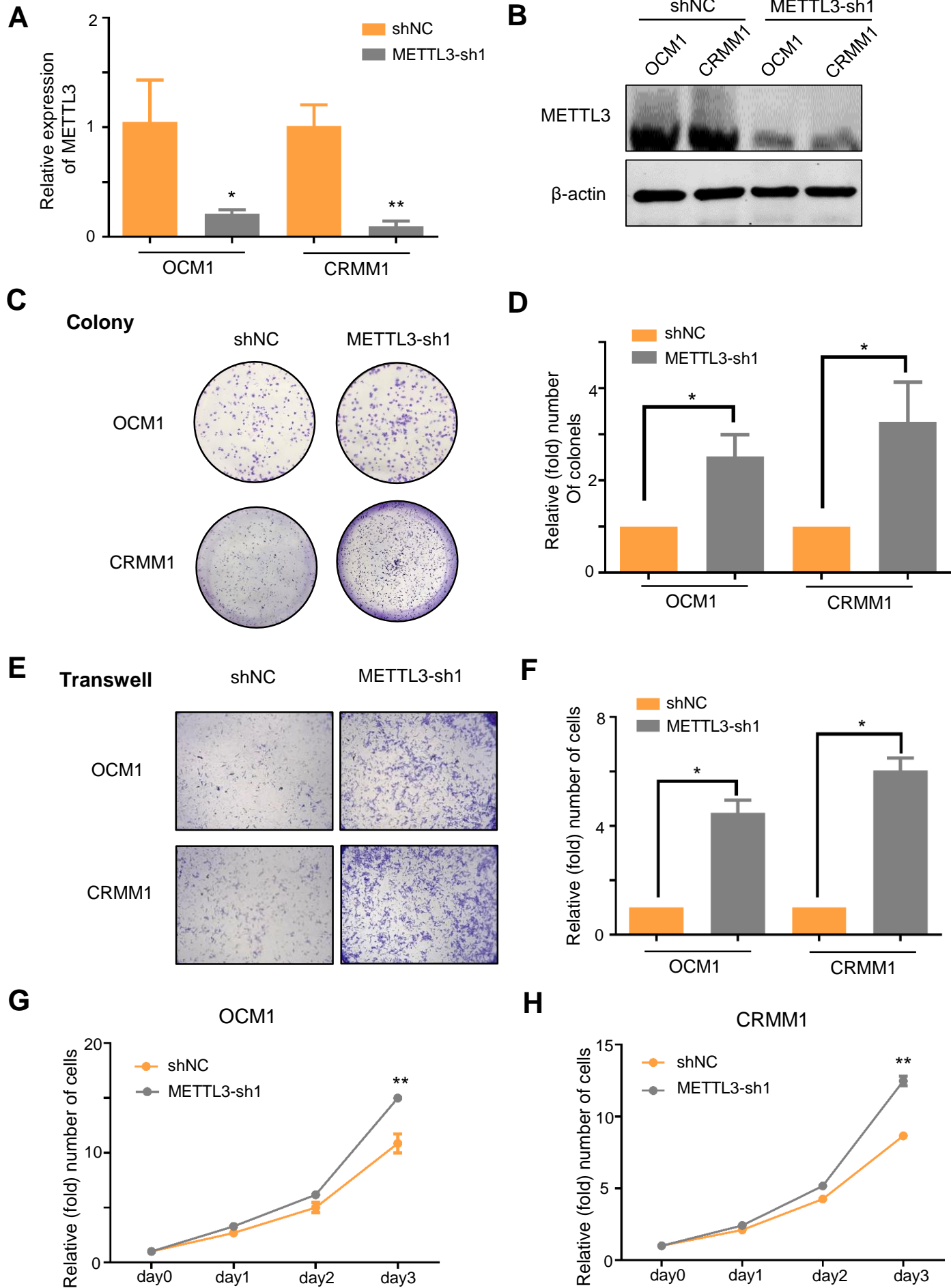

Supplement: Supplementary file 5 — Additional file 5: Figure S2. Lower m6A methylation promoted ocular melanoma tumorigenesis. [file 12943_2019_1088_MOESM5_ESM.pdf]

# Additional file 6: **Figure S3.**

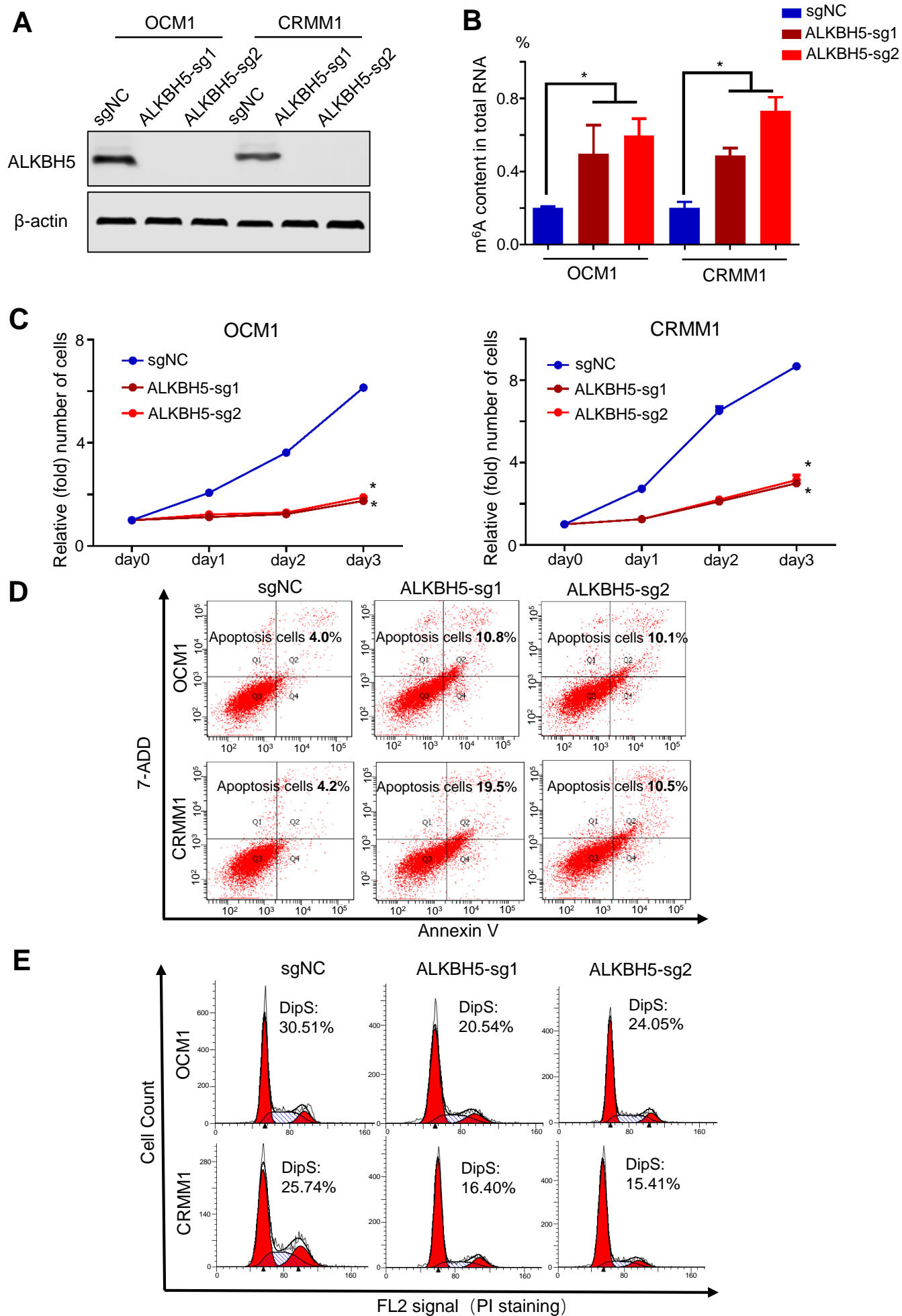

Supplement: Supplementary file 6 — Additional file 6: Figure S3. Higher m6A methylation inhibited ocular melanoma tumorigenesis. [file 12943_2019_1088_MOESM6_ESM.pdf]

# Additional file 7: Figure S4.

**A**

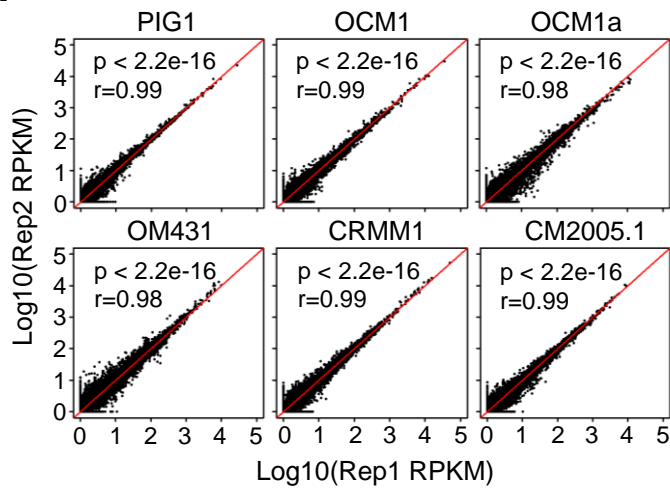

**B**

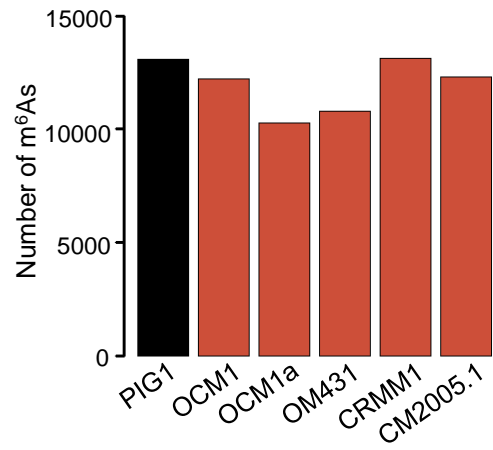

**C**

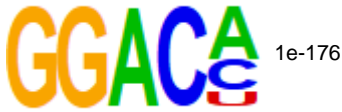

**E**

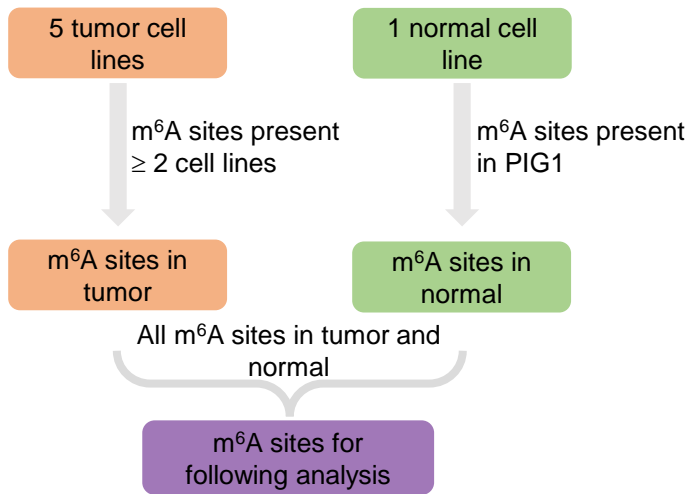

**D**

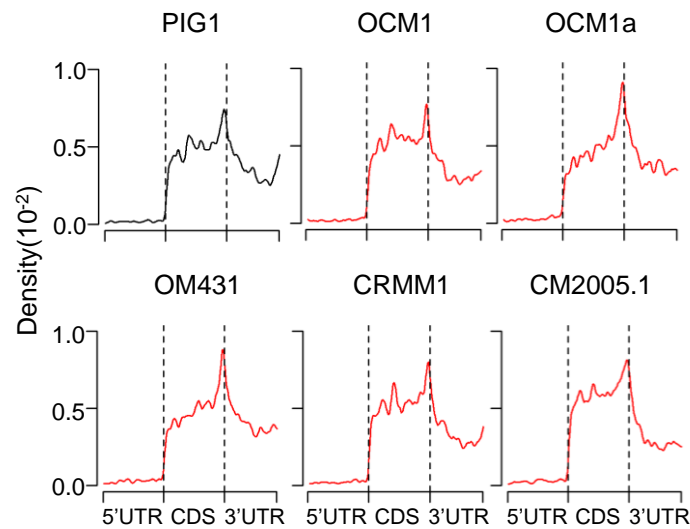

**F**

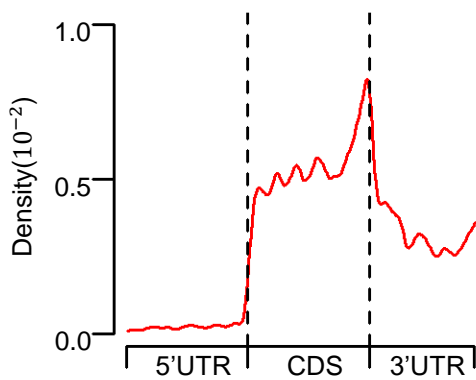

**G**

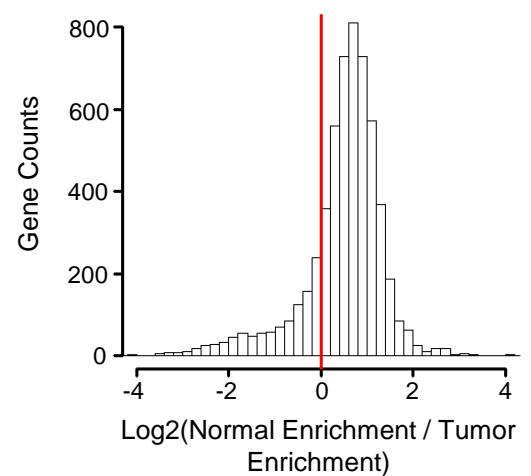

Supplement: Supplementary file 7 — Additional file 7: Figure S4. m6A-seq of ocular melanoma and normal control cells. [file 12943_2019_1088_MOESM7_ESM.pdf]

Additional file 8: **Figure S5.**

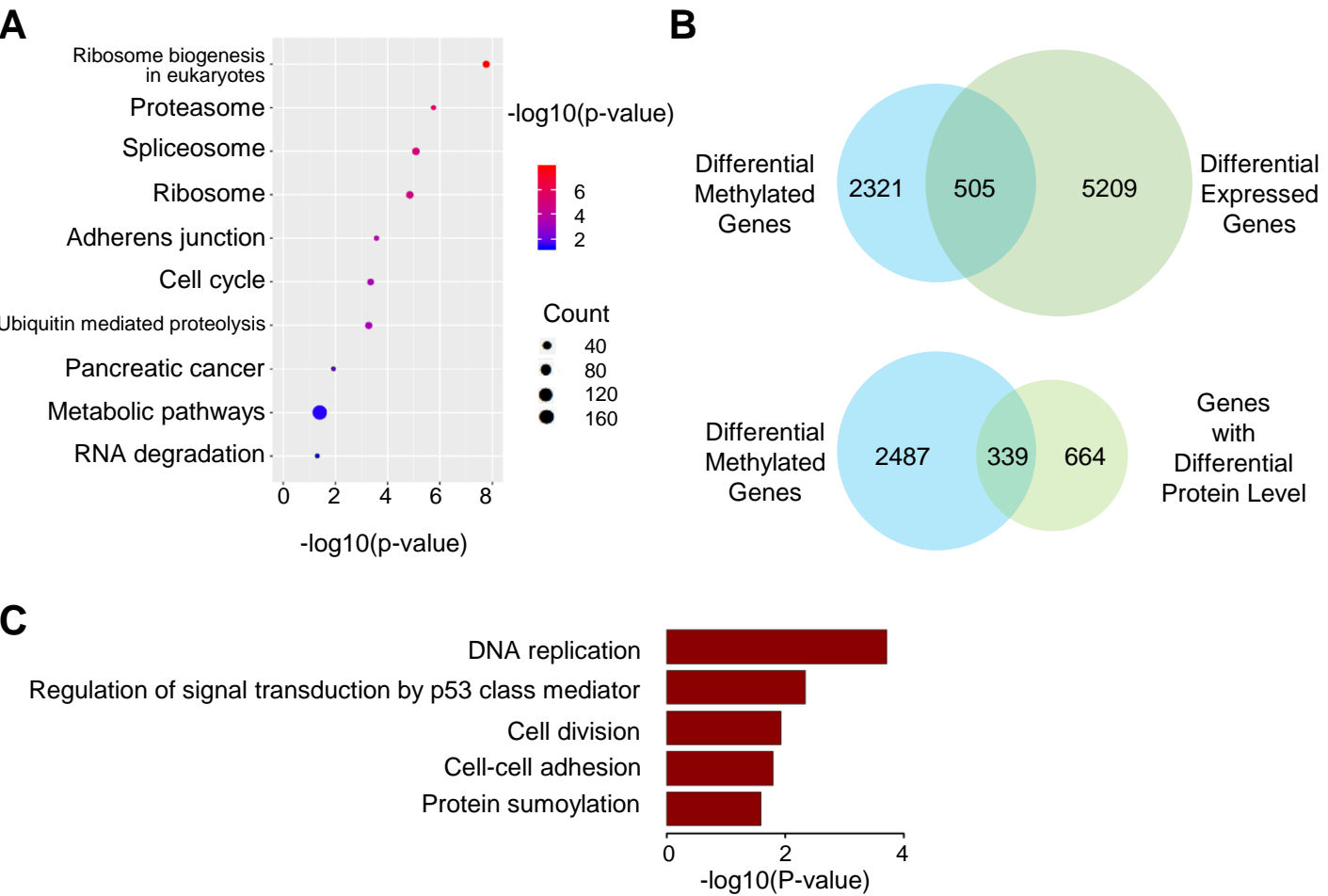

Supplement: Supplementary file 8 — Additional file 8: Figure S5. Characterization and modulation of m6A in ocular melanoma cells. [file 12943_2019_1088_MOESM8_ESM.pdf]

# Additional file 10: Figure S6.

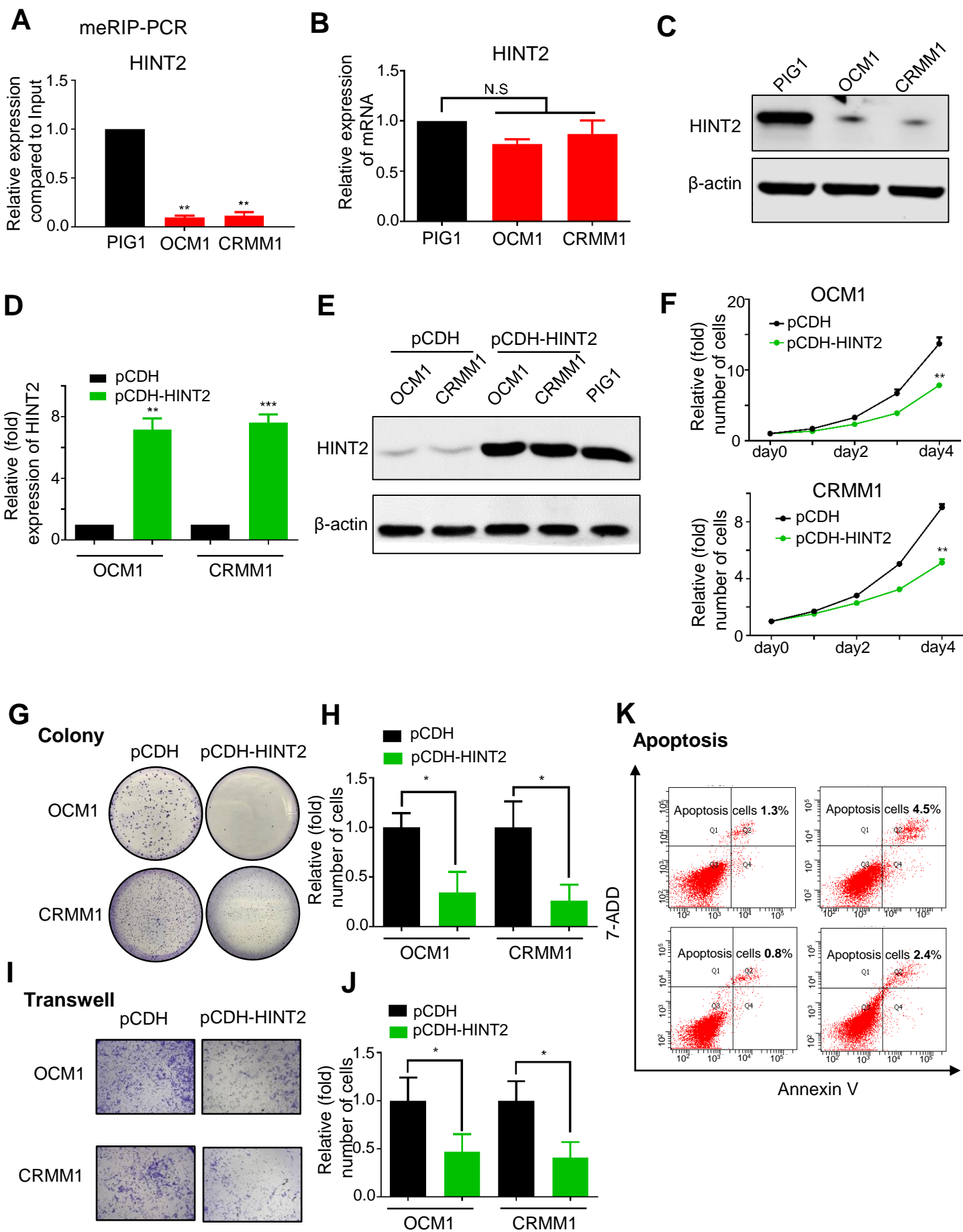

Supplement: Supplementary file 10 — Additional file 10: Figure S6. Overexpression of HINT2 inhibited ocular melanoma tumorigenesis. [file 12943_2019_1088_MOESM10_ESM.pdf]

Additional file 11: **Figure S7.**

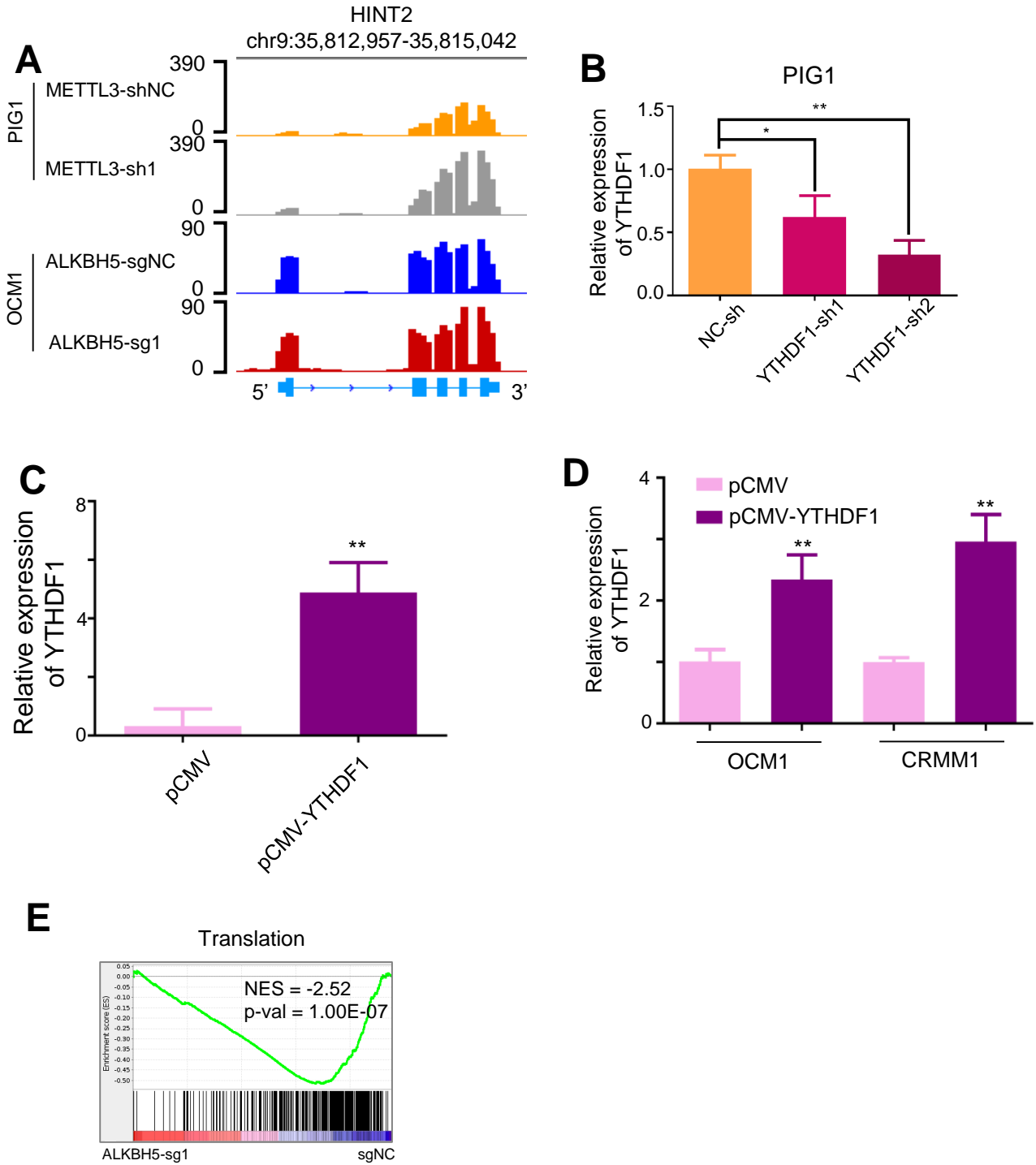

Supplement: Supplementary file 11 — Additional file 11: Figure S7. HINT2 translation was promoted by m6A modification. [file 12943_2019_1088_MOESM11_ESM.pdf]

Fig1I

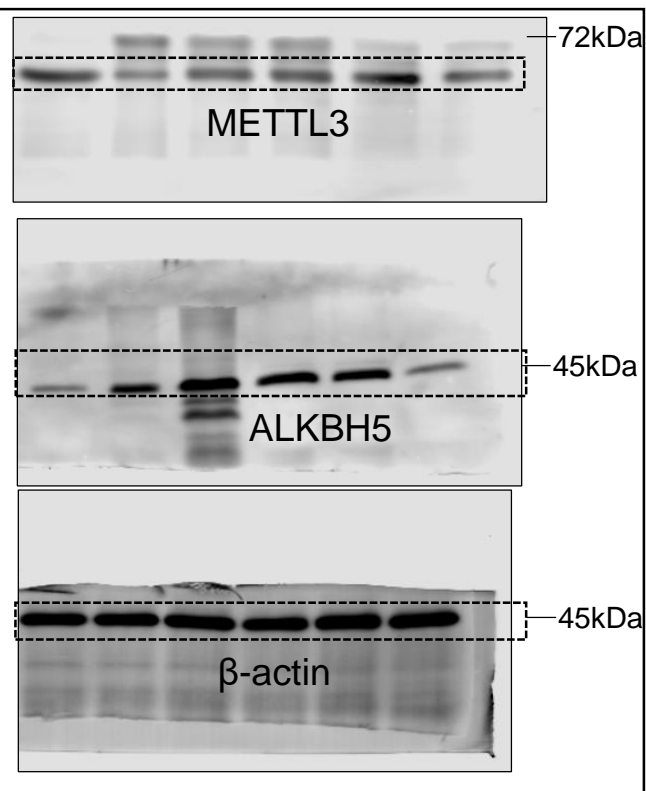

Fig6E

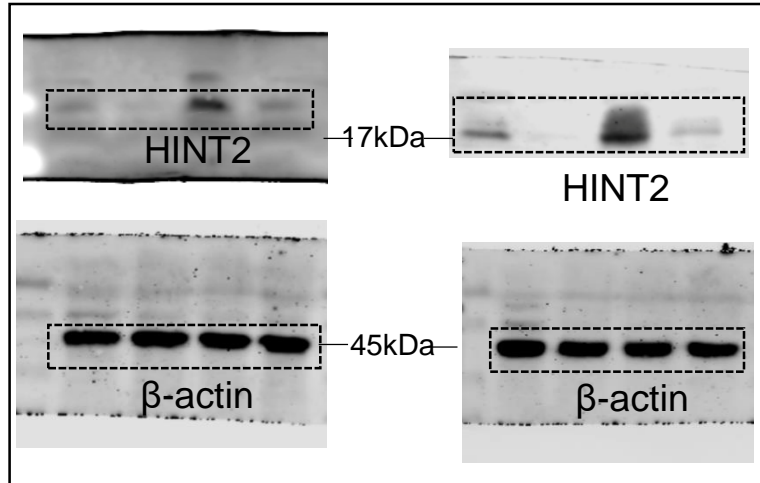

Fig7C

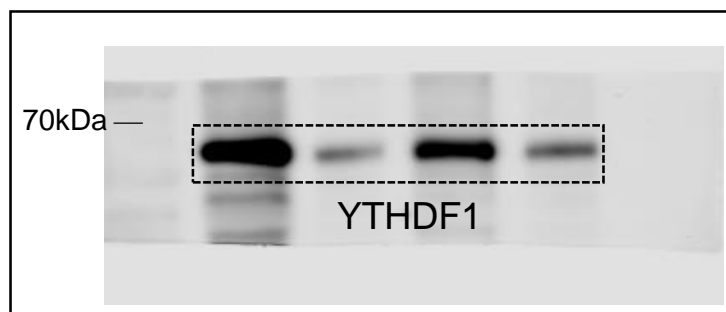

Fig6C

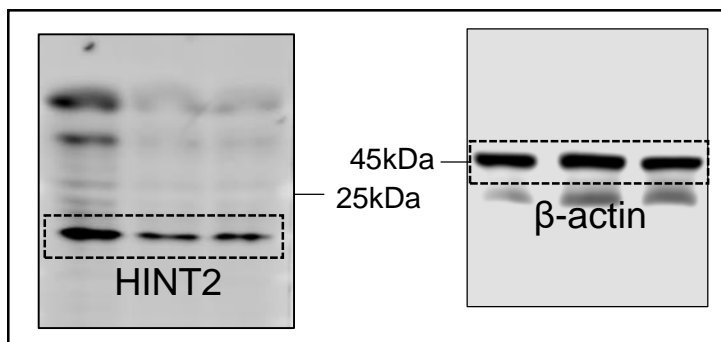

Fig7C

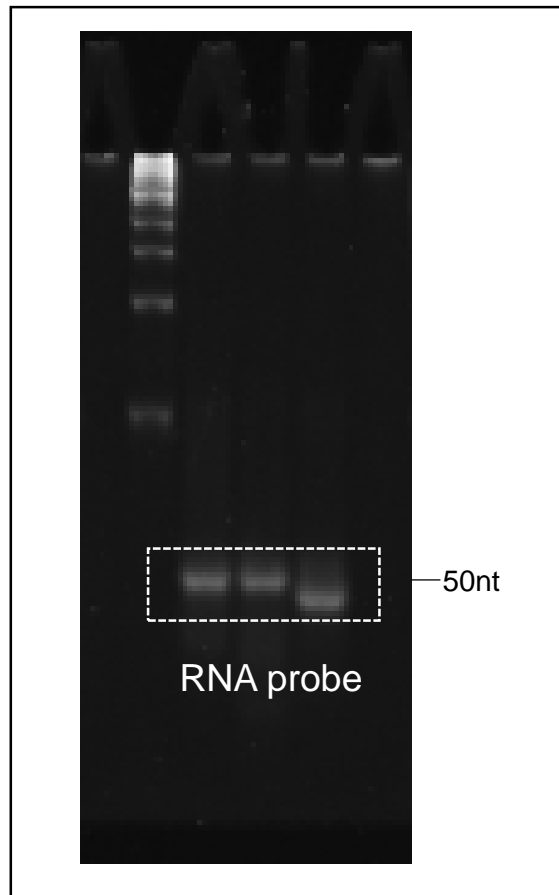

Fig6D

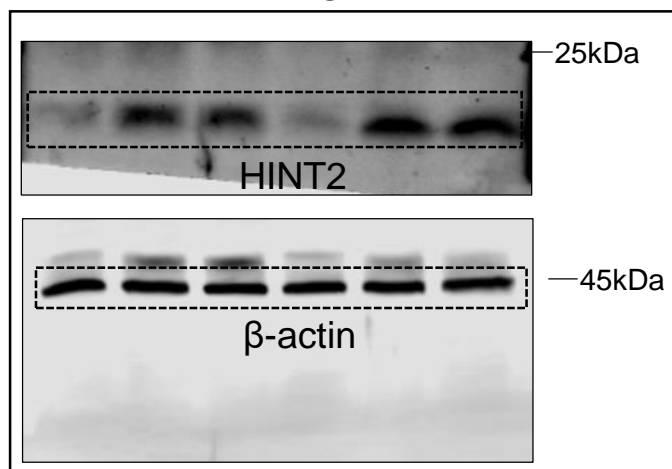

Fig7D

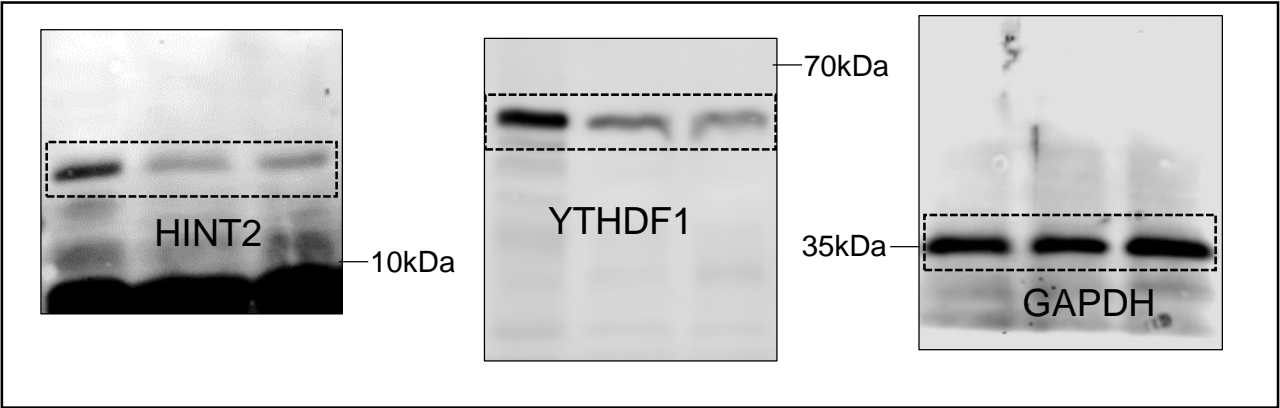

Fig7E

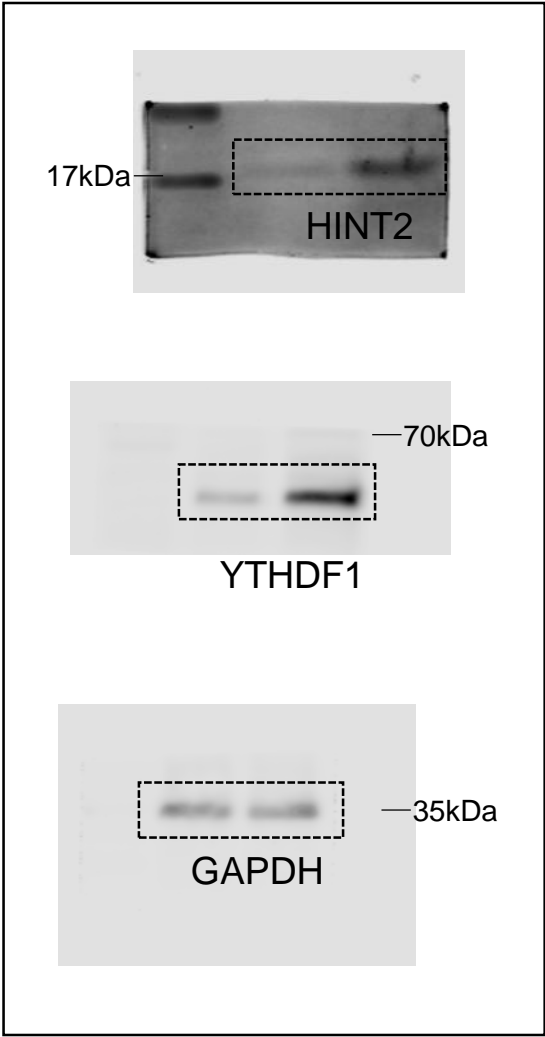

Fig7F

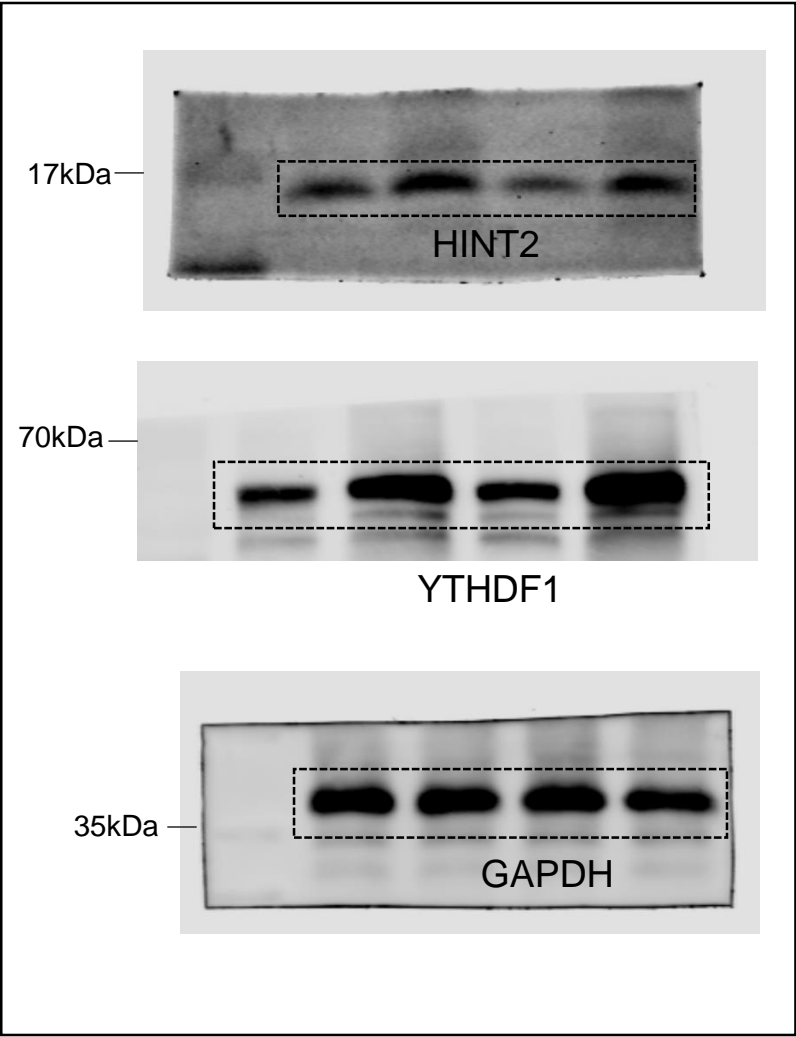

Fig S1B

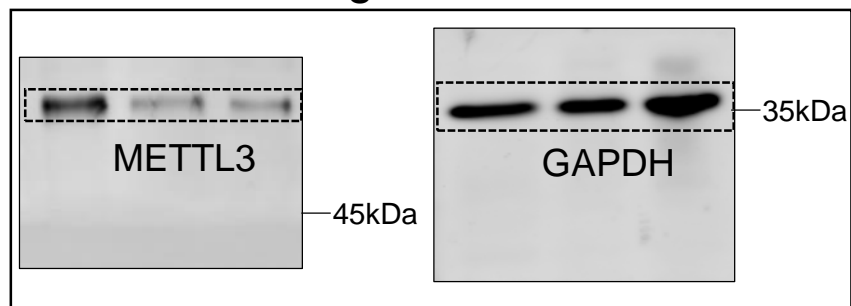

Fig S2B

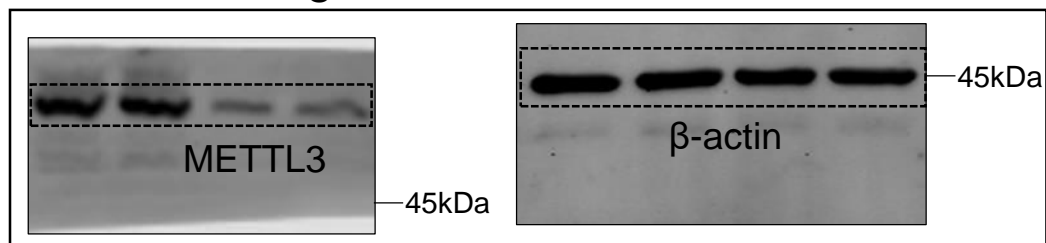

Fig S3A

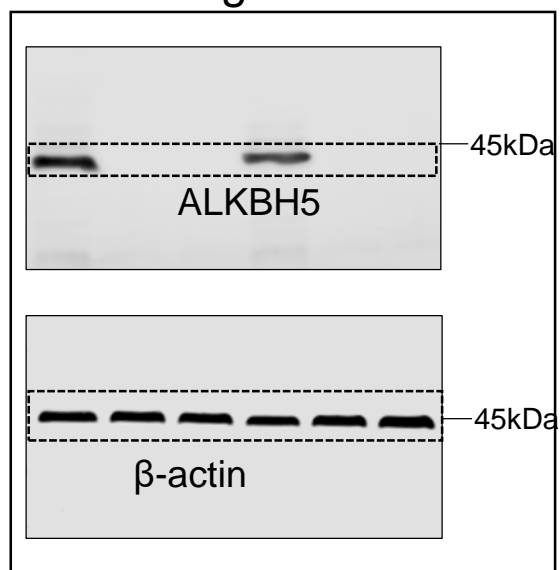

Fig S6C

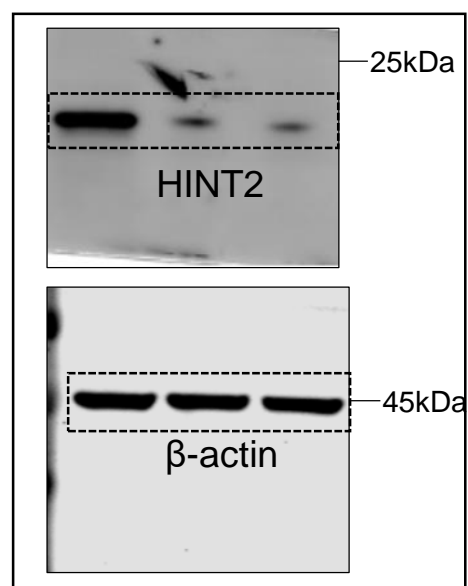

Fig S6E

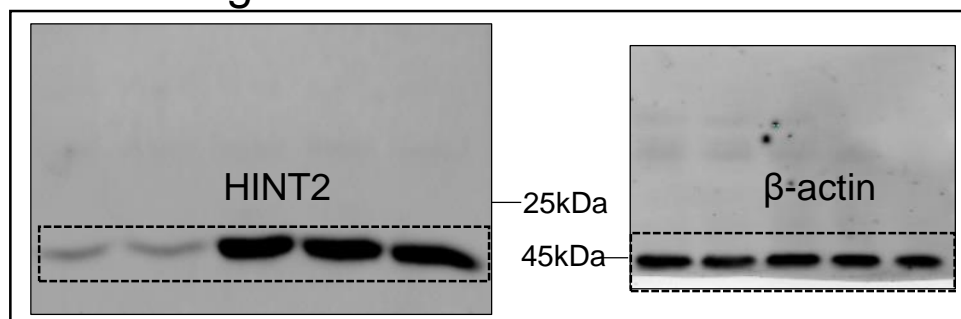

Supplement: Supplementary file 15 — Additional file 15. Unprocessed original scans of blots. [file 12943_2019_1088_MOESM15_ESM.pdf]
