# Supplementary material for: m6A modification suppresses ocular melanoma through modulating HINT2 mRNA translation
Source: Mol Cancer. 2019 Nov 14;18:161. doi: 10.1186/s12943-019-1088-x (PMC6854757; doi:10.1186/s12943-019-1088-x)
Supplement: Supplementary file 12 — Additional file 12: Table S5. Oligonucleotides used in this study. [file 12943_2019_1088_MOESM12_ESM.pdf]

Additional file 12: **Table S5.** Oligonucleotides used in this study

Oligonucleotides used for cloning sgRNA expression vector

**Oligonucleotides name**	**Sequence (5’-3’)**
ALKBH5-sgRNA-1-forward	CACCGGGGATCTCGTCCACGTCGCC
ALKBH5-sgRNA-1-reverse	AAACGGCGACGTGGACGAGATCCCC
ALKBH5-sgRNA-2-forward	CACCGGACGTCCCGGGACAACTATA
ALKBH5-sgRNA-2- reverse	AAACTATAGTTGTCCCGGGACGTCC

Oligonucleotides used for cloning shRNA expression vector

**Oligonucleotides name**	**Sequence (5’-3’)**
METTL3-shRNA-1-forward	GCAAGTATGTTCACTATGAAA
METTL3-shRNA-1-reverse	TTTCATAGTGAACATACTTGC
METTL3-shRNA-2-forward	GCTGCACTTCAGACGAATTAT
METTL3-shRNA-2-reverse	ATAATTCGTCTGAAGTGCAGC
YTHDF1-shRNA-1-forward	GTTCGTTACATCAGAAGGATA
YTHDF1-shRNA-1-reverse	TATCCTTCTGATGTAACGAAC
YTHDF1-shRNA-2-forward	CCCGAAAGAGTTTGAGTGGAA
YTHDF1-shRNA-2-reverse	TTCCACTCAAACTCTTTCGGG

Oligonucleotides used for siRNA

**Oligonucleotides name**	**Sequence (5’-3’)**
HINT2-siRNA-1	TGCACAATCTGTGTATCATCTGC
HINT2-siRNA-2	GTGTATCATCTGCACATTCATGT

ssRNA probes used for RNA pull-down

**Oligonucleotides name**	**Sequence (5’-3’)**
A probe	TGAACCTGCCAACTGATTAAAGGACACCAGACT CTGGATGCTTGGATGGA-Biotin
m^6A probe	TGAACCTGCCAACTGATTAAAGGACACCAGm^6A CTCTGGATGCTTGGATGGA-Biotin
UTR-NC probe	AAGGGAAAAATGGACCCTGTGATGCTAATAAAA CTGTTCTCCCTTAA-Biotin

Oligonucleotides used for luciferase reporter gene

**Oligonucleotides name**	**Sequence (5’-3’)**
HINT2-3’UTR-WT	ACCTGCCAACTGATTAAAGGACACC AGACTCTGGATGCTTGGATGGAAAG GGAAAAATGGACCCTGTGATGCTAA TAAAACTGTTCTCCCTTAA
HINT2-3’UTR-MT	ACCTGCCAACTGATTAAAGGACACC AGTCTCTGGATGCTTGGATGGAAAG GGAAAAATGGACCCTGTGATGCTAA TAAAACTGTTCTCCCTTAA
