# Supplementary material for: m6A modification suppresses ocular melanoma through modulating HINT2 mRNA translation
Source: Mol Cancer. 2019 Nov 14;18:161. doi: 10.1186/s12943-019-1088-x (PMC6854757; doi:10.1186/s12943-019-1088-x)
Supplement: Supplementary file 13 — Additional file 13: Table S6. Antibodies used in this study. [file 12943_2019_1088_MOESM13_ESM.pdf]

Additional file 13: **Table S6.** Antibodies used in this study

**Antibodies**	**Catalogue name**	**Company**
Rabbit-anti-N^6-methyladenosine	ABE572	Merck
Rabbit anti-METTL3	ab195352	Abcam
Rabbit anti-ALKBH5	ab195377	Abcam
Rabbit anti-HINT2	ab220935, ab128677	Abcam
Rabbit anti-YTHDF1	17479-1-AP	Proteintech
Rabbit anti-YTHDF2	24744-1-AP	Proteintech
Rabbit anti-YTHDF3	25537-1-AP	Proteintech
Mouse anti-GAPDH	97166	CST
Mouse anti-β-actin	3700	CST
Rabbit IgG		Merck
Anti-Rabbit-IgG-DyLight800	5151	CST
Anti-Rabbit-IgG-Alexa Fluor® 555	4417	CST
Anti-Mouse-IgG-DyLight800	5257	CST
