# Supplementary material for: m6A modification suppresses ocular melanoma through modulating HINT2 mRNA translation
Source: Mol Cancer. 2019 Nov 14;18:161. doi: 10.1186/s12943-019-1088-x (PMC6854757; doi:10.1186/s12943-019-1088-x)
Supplement: Supplementary file 14 — Additional file 14: Table S7. Primers used in this study. [file 12943_2019_1088_MOESM14_ESM.pdf]

Additional file 14: **Table S7.** Primers used in this study

qRT-PCR Primers

**Primer name**	**Sequence (5’-3’)**
HINT2-RIP-forward	GAAGCTGGGTGCACAATCTG
HINT2-RIP-reverse	CCCTTTCCATCCAAGCATCCA

m^6A-RIP-qPCR Primers

**Primer name**	**Sequence (5’-3’)**
METTL3-forward	TTGTCTCCAACCTTCCGTAGT
METTL3-reverse	CCAGATCAGAGAGGTGGTGTAG
ALKBH5-forward	TCAAGCCTATTCGGGTGTCG
ALKBH5-reverse	TTGGGTTTCAGAGCAGGGTC
HINT2-forward	CTTGTGTTCCGTGATGTGGC
HINT2-reverse	ACAGATTGTGCACCCAGCTT
YTHDF1-forward	CGTGGACACCCAGAGAACAA
YTHDF1-reverse	TGCCCAAAAACAGCATCGTG
